# Advancing therapeutic frontiers: a pipeline of novel drugs for UC management

**DOI:** 10.3389/fgstr.2026.1747118

**Published:** 2026-01-30

**Authors:** Luisa Bertin, Alessandro Massano, Carlo Redavid, Marco Scarpa, Cesare Ruffolo, Imerio Angriman, Andrea Buda, Fabiana Zingone, Brigida Barberio, Edoardo Vincenzo Savarino

**Affiliations:** 1 Gastroenterology Unit, Department of Surgery, Oncology and Gastroenterology, University of Padova, Padova, Italy; 2 Chirurgia Generale 3 Unit, Azienda Ospedale Università di Padova, Padua, Italy; 3 Gastroenterology Unit, Department of Oncological Gastrointestinal Surgery, S. Maria del Prato Hospital, Feltre, Italy

**Keywords:** biologic therapy, clinical trials, combination therapy, inflammatory bowel disease, JAK inhibitors, TL1A pathway, TYK2 inhibitors, UC

## Abstract

Ulcerative colitis is a chronic inflammatory bowel disease with rising global prevalence. Despite therapeutic advances including biologic agents targeting tumor necrosis factor-alpha, integrins, and interleukin pathways, alongside Janus kinase inhibitors and sphingosine-1-phosphate receptor modulators, substantial unmet needs persist in moderate to severe disease. Current advanced therapies achieve clinical response rates of only 30-60% in trials, with approximately 20% of patients requiring hospitalization and 7% undergoing colectomy within five years of diagnosis. The therapeutic pipeline for moderate to severe ulcerative colitis currently encompasses over 100 investigational agents in Phase II and III clinical development. Emerging mechanisms include next-generation Janus kinase and tyrosine kinase 2 inhibitors with enhanced selectivity, novel cell trafficking modulators, advanced tumor necrosis factor-alpha inhibition strategies, and selective interleukin-23 pathway antagonists. Tumor necrosis factor-like ligand 1A pathway inhibitors demonstrate particularly robust efficacy in early trials, with clinical remission rates exceeding 25% compared to less than 2% for placebo. Additional promising approaches target immune checkpoint pathways, receptor-interacting protein kinase 1, and intracellular signaling cascades. innovative combination therapy approaches demonstrated to achieve superior response rates compared to monotherapy. The convergence of novel therapeutic targets, gut-selective compounds minimizing systemic immunosuppression, and biomarker-guided therapy selection represents a paradigm shift toward precision medicine. These advances hold genuine promise for transforming moderate to severe ulcerative colitis management.

## 1 Introduction

Ulcerative colitis (UC) is a chronic inflammatory bowel disease (IBD) characterized by continuous mucosal inflammation extending from the rectum proximally through the colon (1, 2). Its global prevalence is on the rise (3) bu despite significant therapeutic advances over the past two decades, substantial unmet medical needs persist in UC management.

Beyond its physical manifestations, UC profoundly impacts quality of life and contributes to substantial disability. Sleep disturbances affect a significant proportion of patients and correlate with disease activity (4), while factors including disease phenotype, extraintestinal manifestations, and psychological distress contribute to overall disability burden (5, 6). Validated instruments such as the IBD-disk demonstrate the multidimensional nature of disability in UC, encompassing physical, psychological, and social domains (7). Furthermore, severe fatigue, often associated with sarcopenia, anxiety, and systemic inflammation, represents a major contributor to impaired quality of life (8). The adaptation of quality of care standards emphasizes the importance of comprehensive disease management addressing both clinical and patient-reported outcomes (9). Despite significant therapeutic advances over the past two decades, substantial unmet medical needs persist in UC management. The prevalence of anxiety and depression in IBD patients is considerably elevated, with systematic reviews demonstrating that approximately one-third of patients experience clinically significant psychological symptoms, further compounding disease burden (10).

**Figure 1:**
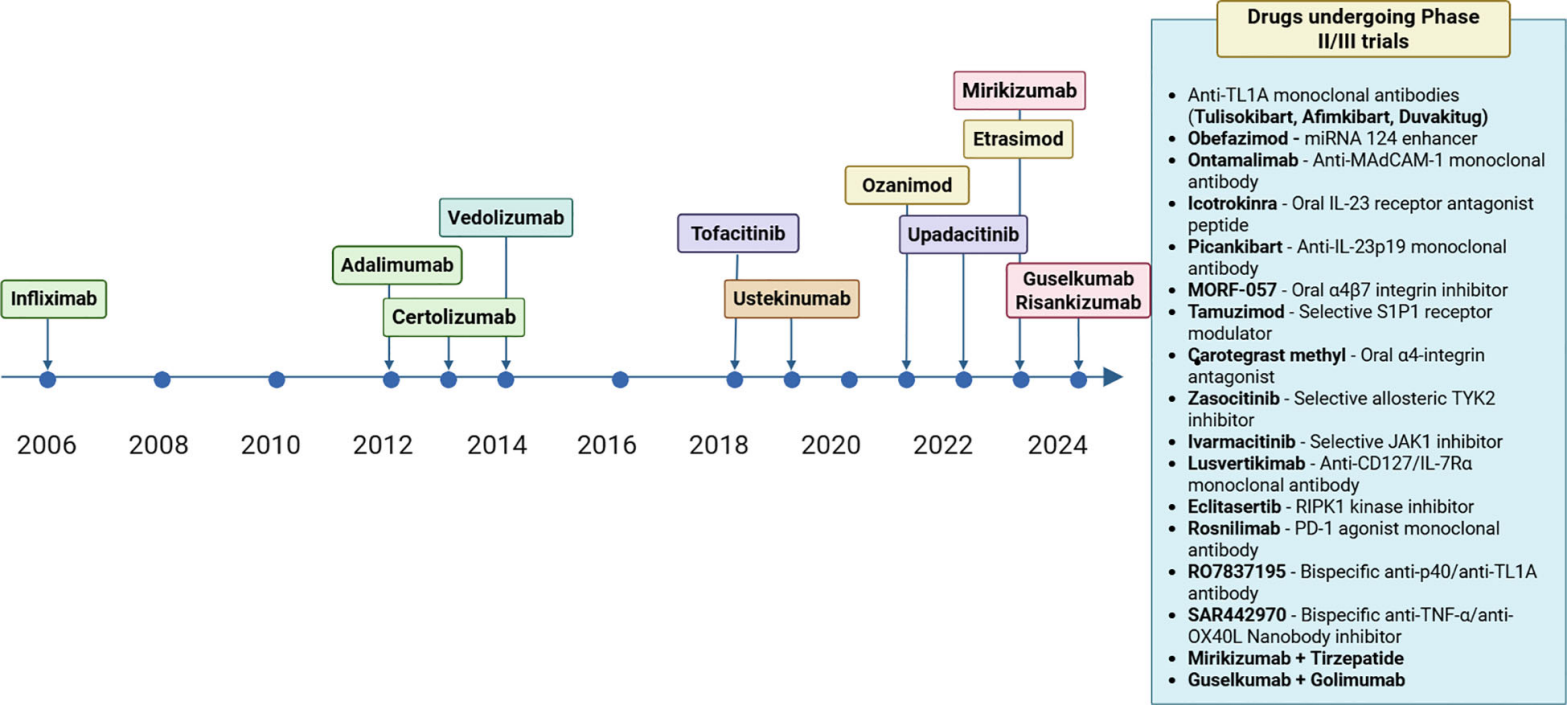
Timeline of FDA-approved FDA therapies for ulcerative colitis. Created with Biorender.com.

Current therapeutic approaches for UC follow a stepped-care model based on disease severity and location. First-line therapy for mild to moderate disease relies on 5-aminosalicylic acid compounds, while moderate to severe disease often requires systemic corticosteroids as bridging therapy to more definitive treatments (11, 12). The introduction of biologic therapies targeting tumor necrosis factor-α (TNF-α) (infliximab, adalimumab, golimumab), integrin α4β7 (vedolizumab), and interleukin (IL)-12/23 pathways (ustekinumab, mirikizumab, guselkumab, risankizumab) has transformed treatment paradigms for moderate to severe UC (11, 13–21). More recently, oral small molecule therapies including Janus kinase (JAK) inhibitors (tofacitinib, upadacitinib, filgotinib) and sphingosine-1-phosphate receptor (S1PR) modulators (ozanimod, etrasimod) have expanded therapeutic options (22–26). **Figure 1** presents a comprehensive timeline of all FDA-approved therapies for ulcerative colitis from 2006 to 2024, incorporating recent corrections to approval dates and highlighting the emergence of novel therapeutic classes.

**Figure 2:**
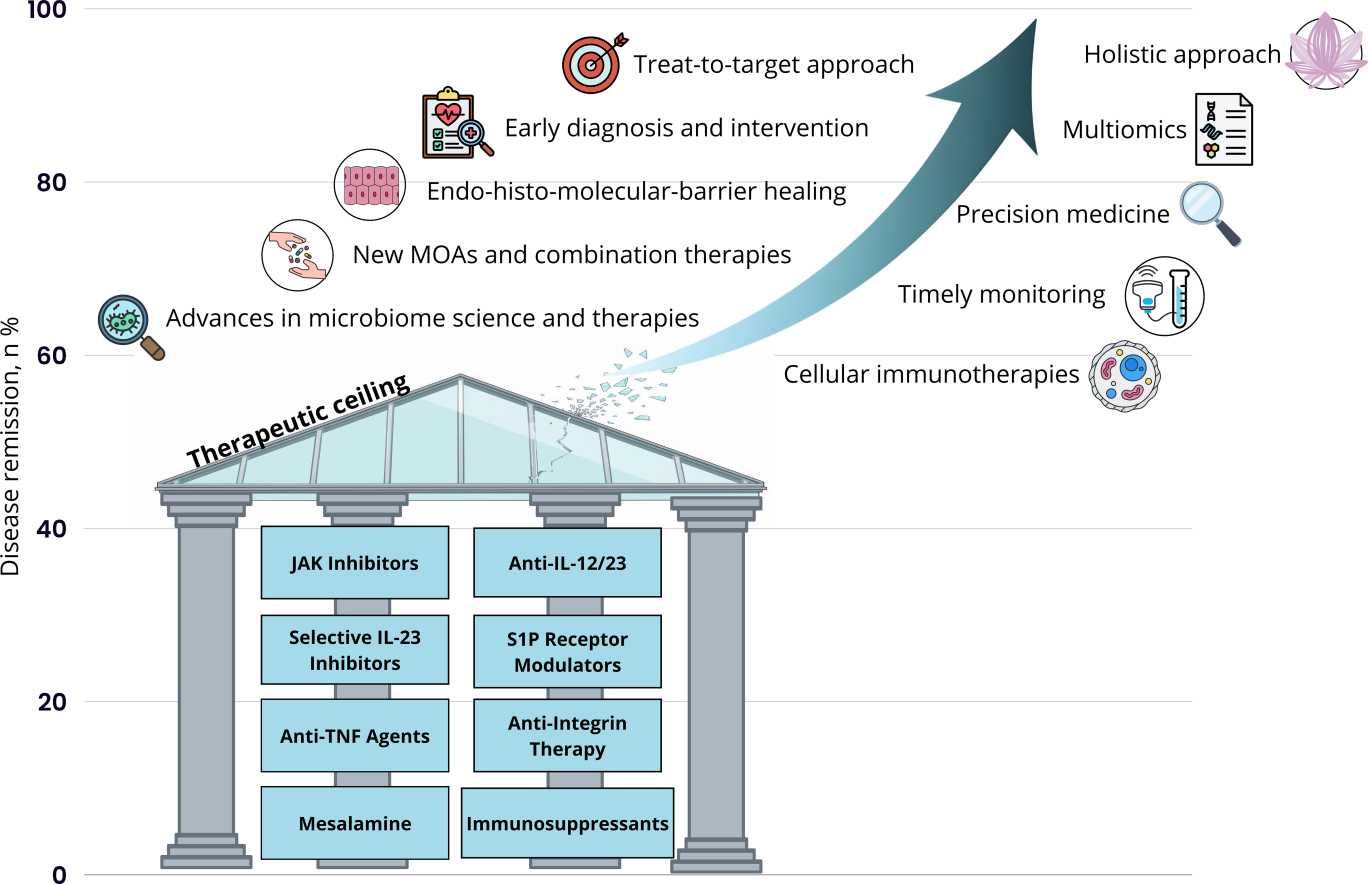
Breaking the therapeutic ceiling in ulcerative colitis. ‘conceptual framework adapted from Raine T, Danese S (27).

However, significant limitations remain with existing treatments. Clinical response rates for advanced therapies typically range from 30% to 60% in clinical trials, with effectiveness declining with successive treatment failures (27). Primary non-response, secondary loss of response, and treatment-limiting adverse events (AEs) continue to affect substantial proportions of patients. Within five years of diagnosis, approximately 20% of UC patients require hospitalization and 7% undergo colectomy, highlighting the persistent morbidity associated with this condition (28, 29). The risk of colorectal cancer (CRC) remains elevated, with a 1.7-fold higher risk compared to the general population and cumulative cancer risk reaching 4.5% after 20 years of disease duration (30). **Figure 2** illustrates the concept of the therapeutic ceiling in ulcerative colitis (UC), representing the plateau in clinical remission rates achieved with current treatment modalities. Emerging therapies, particularly selective IL-23 inhibitors and JAK inhibitors, appear to exceed this threshold, suggesting the potential to “break the therapeutic ceiling” and achieve improved long-term outcomes for patients with UC (27). Beyond improving efficacy outcomes, there remains an equally pressing need for novel therapeutic agents with improved safety and tolerability profiles. Current advanced therapies are associated with various safety concerns. The ideal next-generation therapies should not only break through the therapeutic ceiling but also offer favorable benefit-risk profiles suitable for long-term maintenance therapy.

The therapeutic landscape for UC is rapidly evolving, with an unprecedented number of investigational agents currently in clinical development. This pipeline encompasses novel mechanisms of action targeting diverse inflammatory pathways, including advanced TNF-α inhibition strategies, next-generation JAK and Tyrosine Kinase 2 (TYK2) inhibitors, cell trafficking modulators, IL-23 pathway antagonists, and innovative approaches targeting costimulation, checkpoint pathways, and tissue repair mechanisms. Moreover, it includes drugs like obefazimod which enhances the expression of the anti-inflammatory microRNA, miR-124. Additionally, combination therapy strategies represent emerging paradigms that may enhance therapeutic efficacy.

This comprehensive review examines the current pipeline of investigational therapies for moderate to severe UC across Phase II and III of clinical development. We systematically analyze emerging drugs by therapeutic class and mechanism of action, evaluate novel combination strategies, and discuss the potential impact of these advances on future UC management. The investigational agents currently in Phase II and III clinical development for moderate to severe UC are summarized in **Table 1**, which classifies these emerging therapies by mechanism of action, specific molecular targets, and current trial status. This review focuses on therapeutic agents currently in active development for UC. However, to provide context and highlight the high rate of attrition observed in UC drug development, agents that have been studied historically but have not progressed further are listed in **Table 1**. Understanding this therapeutic pipeline is crucial for clinicians, researchers, and patients as we move toward more personalized and effective treatment approaches for this challenging chronic inflammatory condition.

**Table 1:** Emerging therapeutic agents for moderate to severe ulcerative colitis: classification, mechanisms, and clinical trial status.

Classification	Drug name	Mechanism of action	Trial name/NCT	Trial phase	Trial status
JAK Inhibitor	Ivarmacitinib (SHR0302)	Selective JAK1 inhibitor blocking JAK-STAT pathway	NCT03675477, NCT05181137	Phase 2, Phase 3	Phase 2 completed Feb 2021; Phase 3 status unknown
JAK Inhibitor	Itacitinib (INCB039110)	JAK1 inhibitor	NCT03627052	Phase 2	Withdrawn Nov 2019 due to lack of recruitment
JAK Inhibitor	Peficitinib (JNJ-54781532)	Oral pan-JAK inhibitor	NCT01959282	Phase 2b	Terminated Aug 2022 due to recruitment difficulties; development discontinued
JAK Inhibitor	Izencitinib (TD-1473)	Gut-selective pan-JAK inhibitor	NCT03758443 (RHEA), NCT03920254	Phase 2b/3	Terminated Oct 2021 based on interim analysis
JAK Inhibitor	Oncostellae (OST-122)	Oral gut-restricted JAK3/TYK2/ARK5 inhibitor	NCT04353791	Phase 1b/2a	Completed Dec 2022; development not pursued further
JAK Inhibitor	Ritlecitinib (PF-06651600)	Oral selective JAK3/TEC family kinase inhibitor	NCT02958865 (VIBRATO)	Phase 2b	Completed May 2021; prioritized for alopecia areata
JAK Inhibitor	Brepocitinib (PF-06700841)	Oral dual TYK2/JAK1 inhibitor	NCT02958865 (VIBRATO)	Phase 2b	Completed May 2021; advanced to alopecia areata trials
TYK2 Inhibitor	Deucravacitinib	Oral selective allosteric TYK2 inhibitor	NCT03934216 (LATTICE-UC), NCT04613518 (IM011-127)	Phase 2	Completed 2021-2023; terminated early for failing primary endpoints
TYK2 Inhibitor	Zasocitinib (TAK-279)	AI-designed highly selective allosteric TYK2 inhibitor	NCT06254950, NCT06764615	Phase 2	Recruiting; estimated completion Aug 2027
TYK2 Inhibitor	Ropsacitinib (PF-06826647)	Selective TYK2 inhibitor	NCT04209556	Phase 2b	Withdrawn with zero enrollment due to portfolio re-prioritization
TYK2 Inhibitor	D-2570	Novel oral TYK2 inhibitor	NCT07035041	Phase 2	Recruiting; began May 2025, estimated completion Dec 2026
α4-integrin antagonist	Carotegrast methyl (AJM300)	Oral small-molecule α4-integrin antagonist	NCT03531892	Phase 3	Completed; 45% clinical response vs 21% placebo at week 8
Integrin antagonist	Milategrast (E6007)	Small molecule integrin antagonist blocking calreticulin-integrin interaction	NCT03018054	Phase 2	Completed Aug 2019; development discontinued May 2022
α4β7 antagonist	PTG-100	Oral α4β7 integrin antagonist peptide	NCT02895100	Phase 2b	Terminated early for futility March 2018
α4β7 antagonist	PN-943	Oral gastrointestinal-restricted cyclic peptide targeting α4β7	NCT04504383	Phase 2	Completed Feb 2023; primary endpoint not met for 450mg dose
α4β7 antagonist	Abrilumab	Human monoclonal IgG2 antibody targeting α4β7 integrin	NCT01694485, NCT01959165	Phase 2b	Global study completed; 13.3% remission (70mg) vs 4.3% placebo
α4β7 antagonist	Vatelizumab (SAR339658)	Monoclonal antibody targeting α4β7 integrin	NCT01659138, NCT01861249	Phase 2	Terminated early due to recruitment challenges
β7 integrin antagonist	Etrolizumab	Gut-targeted anti-β7 integrin monoclonal antibody	Multiple NCT numbers	Phase 2/3	Program terminated 2023; failed to show benefit vs comparators
α4β7 antagonist	Emvistegrast (GS-1427)	Oral α4β7 integrin inhibitor	NCT06290934	Phase 2	Primary completion Oct 2025, final completion Feb 2027
α4β7 antagonist	MORF-057	Oral small-molecule inhibitor of α4β7 integrin	NCT05291689 (EMERALD-1), NCT05611671 (EMERALD-2)	Phase 2a, Phase 2b	EMERALD-1 completed Nov 2023; EMERALD-2 primary completion Nov 2024
MAdCAM-1 antagonist	Ontamalimab	Fully human IgG2 monoclonal antibody binding MAdCAM-1	NCT03259334 (FIGARO UC 301/302), NCT03290781 (FIGARO UC 303)	Phase 3	Completed; both 25mg and 75mg doses showed significant efficacy
S1P1 modulator	Icanbelimod (CBP-307)	Selective small-molecule S1P1 receptor modulator	NCT04700449	Phase 2	Completed Nov 2022; primary endpoint not met but secondary endpoints showed signals
S1P modulator	LC51-0255	Oral S1P modulator	NCT04096573	Phase 2	Withdrawn with zero enrollment due to company decision
S1P modulator	Mocravimod (KRP203)	Oral S1PR modulator	NCT01375179	Phase 2	Terminated early May 2012 after enrolling only 27 of 72 participants
S1P1 modulator	Tamuzimod (VTX002)	Selective sphingosine 1-phosphate receptor 1 modulator	NCT05156125	Phase 2	Completed Aug 2023; 28% clinical remission (60mg) vs 11% placebo
CXCR2 antagonist	Elubrixin (SB-656933)	Orally active selective CXCR2 antagonist and IL-8 receptor antagonist	NCT00748410	Phase 2a	Terminated early after enrolling only 3 participants
PSGL-1 agonist	Leiolizumab	Tetravalent agonist IgG1 antibody targeting PSGL-1	NCT06109441	Phase 2a	Started Dec 2023; estimated completion Nov 2026
PSGL-1 agonist	Neihulizumab (ALTB-168)	IgG4 monoclonal antibody targeting PSGL-1	NCT03298022	Phase 2	Terminated due to COVID-19; completed with 24 patients
IP-10 antagonist	Eldelumab (BMS-936557)	Fully human monoclonal IgG1 antibody targeting IP-10/CXCL10	NCT00656890, NCT01294410	Phase 2, Phase 2b	Completed; failed to meet primary endpoints
CCR9 antagonist	GSK1605786	Oral CCR9 chemokine receptor antagonist	NCT01658605	Phase 2	Terminated with zero enrollment
TNF modulator	Hemay007	Small molecule TNF-α regulator	NCT03977480	Phase 2	Terminated early Aug 2022 due to recruitment difficulties
TNF-α/OX40L inhibitor	SAR442970	Bispecific pentavalent Nanobody targeting TNF-α and OX40L	NCT06975722	Phase 2b	Started July 2025; estimated completion Dec 2026
TNFR1 antagonist	Balinatunfib (SAR441566)	Small molecule stabilizing asymmetric soluble TNF	NCT06867094	Phase 2	Recruiting; started March 2025, estimated completion July 2027
CD40 antagonist	Ravagalimab (ABBV-323)	IgG1 monoclonal antibody antagonizing CD40	NCT03695185	Phase 2a	Completed Sept 2021; used synthetic placebo control
IL-23R antagonist	Icotrokinra	First-in-class targeted oral peptide blocking IL-23 receptor	NCT06049017 (ANTHEM-UC)	Phase 2b	Met primary endpoint; 63.5% response (highest dose) vs 27% placebo
IL-23p19 inhibitor	Picankibart (IBI112)	Recombinant anti-IL-23p19 monoclonal IgG1 antibody	NCT05377580	Phase 2	Primary completion March 2024; met primary efficacy endpoint
IL-23p19 inhibitor	Brazikumab	Fully human IgG2 monoclonal antibody targeting IL-23 p19	NCT03616821, NCT04277546	Phase 2	Terminated Oct 2023 due to strategic decision
IL-13 inhibitor	Anrukinzumab	Humanized monoclonal IgG1 antibody targeting IL-13	NCT01284062	Phase 2a	Completed April 2013; failed to show efficacy
IL-13 inhibitor	Tralokinumab (CAT-354)	Recombinant human monoclonal IgG4 antibody against IL-13	NCT01482884	Phase 2a	Completed June 2013; improved remission rates (18% vs 6%)
IL-4Rα antagonist	Dupilumab	Humanized monoclonal IgG4 antibody binding IL-4 receptor alpha	NCT05731128	Phase 2	Started Jan 2023; estimated completion March 2027
IL-36R antagonist	Spesolimab (BI655130)	Humanized monoclonal IgG1 antibody targeting IL-36 receptor	NCT03482635, NCT03100864, NCT03123120, NCT03648541	Phase 2	Completed; consistently showed lack of efficacy
gp130 antagonist	Olamkicept	gp130 trans-signaling inhibitor	NCT03235752	Phase 2b	Completed Dec 2020; 58.6% response (600mg) vs 34.5% placebo
CD127 antagonist	Lusvertikimab (OSE-127)	First-in-class monoclonal IgG4 antibody targeting CD127 (IL-7Rα)	NCT04882007 (CoTikiS)	Phase 2	Completed Jan 2025; 22% remission (450mg) vs 4.4% placebo
IL-1β inhibitor	Lutikizumab (ABT-981)	Humanized IgG4 monoclonal antibody binding IL-1β	NCT06257875 (Horizon)	Phase 2	Interim analysis July 2025 showed insufficient differentiation vs adalimumab
CD20 antagonist	Rituximab	IgG1 monoclonal antibody targeting CD20 on B lymphocytes	NCT00261118	Phase 2/3	Completed 2009; no significant benefit despite B-cell depletion
CD28 antagonist	Abatacept (Orencia)	CTLA-4-Ig fusion protein blocking CD28-CD80/86	NCT00410410	Phase 3	Completed Nov 2009; failed to show efficacy
OX40 antagonist	KHK4083	Anti-OX40 IgG1 monoclonal antibody	NCT02647866	Phase 2	Completed Oct 2018; well tolerated but no efficacy
PD-1 agonist	Rosnilimab	Novel PD-1 agonist IgG1 monoclonal antibody	NCT06127043 (ROSETTA)	Phase 2	Started Dec 2023; estimated completion May 2026
CD25 antagonist	Basiliximab	Chimeric monoclonal IgG1 antibody targeting CD25	NCT00430898, NCT01061996	Phase 2	Completed Sept 2008; ineffective
LAG-3 antagonist	GSK2831781	Anti-LAG3 cell depleting IgG1 monoclonal antibody	NCT03893565	Phase 2	Terminated early Feb 2021 for futility; no mucosal LAG-3 depletion
OSMRβ antagonist	Vixarelimab	Fully human IgG4 monoclonal antibody targeting OSMRβ	NCT06137183	Phase 2	Terminated June 2025 after futility analysis
TREM-1 antagonist	BI 3032950	TREM-1 antagonist	NCT06636656	Phase 2	Started Dec 2024; estimated completion June 2028
MCR1 agonist	PL-8177	Oral selective melanocortin-1 receptor agonist	NCT05466890	Phase 2a	Completed Feb 2025; 33% remission vs 0% placebo
MMP9 antagonist	Andecaliximab	Recombinant chimeric IgG4 monoclonal antibody inhibiting MMP9	NCT02520284	Phase 2/3	Terminated for futility; no efficacy shown
Interferon	Interferon beta-1a	Type I interferon with immunomodulatory properties, suppresses IL-13 production	Various trials (Nikolaus et al, Musch et al, Pena-Rossi et al)	Phase 2	Completed; failed to show significant benefit vs placebo despite initial pilot data
CCL11 antagonist	Bertilimumab	Recombinant human IgG4 monoclonal antibody neutralizing eotaxin-1	NCT01671956	Phase 2	Status unknown as of Jan 2018; remained as recruiting
PKC inhibitor	Sotrastaurin (AEB071)	Oral pan-protein kinase C inhibitor	NCT00572585	Phase 2	Terminated early for poor efficacy
NF-κB/IRF inhibitor	CU104	Oral drug inhibiting IL-1β, IL-6, TNF-α	NCT05907330	Phase 2	Estimated completion March 2026; not yet recruiting
IL-22 agonist	Efmarodocokin alfa (UTTR1147A)	IL-22 agonist fusion protein	NCT03558152	Phase 2	Completed Dec 2021; terminated for futility
IL-2R agonist	Aldesleukin (ILT-101)	Low-dose IL-2 selectively expanding regulatory T cells	NCT02200445	Phase 1b/2a	Completed March 2021; 69% response at 1.0 million IU/m²/day
IL-2R agonist	NKTR-358 (LY3471851)	PEG-conjugated recombinant IL-2	NCT04677179	Phase 2	Terminated Aug 2022 for enrollment futility
IL-2 mutein	Efavaleukin alfa (AMG 592)	Engineered IL-2 mutein with Fc domain	NCT04987307, NCT05672199	Phase 2, Phase 2 OLE	Terminated Oct 2024 after meeting futility rule
TL1A antagonist	Tulisokibart (MK-7240)	Humanized IgG1 monoclonal antibody targeting TL1A	NCT04996797 (ARTEMIS-UC), NCT06052059, NCT06651281	Phase 2, Phase 3	Phase 2 completed; Phase 3 ongoing, estimated completion Dec 2029
TL1A antagonist	Afimkibart (PF-06480605/RO7790121)	Fully human IgG1 monoclonal antibody targeting TL1A	NCT02840721 (TUSCANY), NCT04090411 (TUSCANY-2), NCT06588855 (Ametrine-2), NCT06589986 (Ametrine-1)	Phase 2a, Phase 2b, Phase 3	Phase 2b completed; Phase 3 recruiting, estimated completion Dec 2029
TL1A antagonist	Duvakitug (TEV-48574)	Fully human IgG1 monoclonal antibody targeting TL1A	NCT05499130 (RELIEVE UCCD), NCT05668013	Phase 2b	Completed Nov 2024; 36-48% remission vs 20% placebo
TL1A antagonist	XmAb942	Anti-TL1A IgG1 antibody with extended half-life	NCT06619990	Phase 1/2	Started Oct 2024; estimated completion Oct 2027
p40/TL1A bispecific	RO7837195	Bispecific antibody targeting IL-12/IL-23 p40 and TL1A	NCT06979336 (SUNCREST)	Phase 2b	Recruiting; first enrollment Q3 2025, estimated completion Oct 2028
RIPK1 inhibitor	GSK2982772	RIPK1 inhibitor	NCT02903966	Phase 2a	Completed 2019; no clinical benefit vs placebo
RIPK1 inhibitor	ABBV-668	Oral inhibitor of RIPK1	NCT05570006	Phase 2	Completed Dec 2024; results not yet reported
RIPK1 inhibitor	Eclitasertib (SAR443122)	Highly potent selective oral RIPK1 kinase inhibitor	NCT05588843 (RESOLUTE)	Phase 2	Started Nov 2022; estimated completion Dec 2026
miRNA upregulation	Obefazimod (ABX464)	Oral small-molecule upregulating miR-124	NCT05507203, NCT05507216 (ABTECT), NCT05535946	Phase 3	Induction trials completed; 50mg achieved 16.4% placebo-adjusted remission
PDE4 inhibitor	Apremilast	Oral phosphodiesterase 4 inhibitor	NCT02289417	Phase 2	Completed June 2019; 31.6% remission (30mg) vs 12.1% placebo
PDE4 inhibitor	Mufemilast (Hemay005)	Selective phosphodiesterase 4 inhibitor	NCT05486104	Phase 2	Started Nov 2022; estimated completion Dec 2025
PDE4 inhibitor	Tetomilast (OPC-6535)	PDE4 inhibitor	NCT00317356	Phase 2	Terminated Aug 2007; did not meet efficacy endpoints
PDE4 inhibitor	Orismilast (UNI-50001)	Oral B/D selective PDE-4 inhibitor	UCORIS trial	Phase 2a	Preliminary results Jan 2025; 33% achieved remission
NLRX1 agonist	Amelenodor	Oral triphenyl compound, NLRX1 agonist	NCT05785715	Phase 2	Terminated early May 2025 after completing enrollment
TLR9 modulator	BL-7040	Synthetic oligonucleotide, TLR9 modulator	NCT01506362	Phase 2a	Completed May 2013; 12.5% remission, good tolerability
Pellino-1 inhibitor	BBT-401-1S	Oral lipidated tetrapeptide inhibiting Pellino-1	NCT04596293, NCT03800420	Phase 2	Completed July 2022; failed to show efficacy
GPR84 antagonist	GLPG1205	Oral selective functional antagonist of GPR84	NCT02337608 (ORIGIN)	Phase 2	Failed to meet efficacy endpoints; development discontinued
LANCL2 agonist	Omilancor (BT-11)	First-in-class oral gut-restricted LANCL2 agonist	NCT03861143	Phase 2	Completed June 2021; 30.4% remission vs 3.7% placebo
LTA4H inhibitor	LYS006	Highly potent selective LTA4H inhibitor	NCT04074590	Phase 2	Terminated Nov 2022 due to strategic considerations
ATPase modulator	Parimifasor (LYC-30937-EC)	Oral enteric-coated ATPase modulator	NCT02762500, NCT02764229	Phase 2	Extension terminated early May 2018 for lack of efficacy
SMAD7 antisense	Mongersen (GED-0301)	21-mer antisense oligonucleotide targeting SMAD7	NCT02601300	Phase 2	Discontinued Oct 2017 due to efficacy concerns and manufacturing issues
MAP3K8/TPL2 inhibitor	Tilpisertib (GS-4875)	Oral MAP3K8/TPL2 inhibitor	NCT04130919, NCT06029972 (PALEKONA)	Phase 2	First study terminated Feb 2021; PALEKONA ongoing, estimated completion 2027
DHODH inhibitor	Vidofludimus calcium (IMU-838)	Oral selective DHODH inhibitor	NCT03341962 (CALDOSE-1)	Phase 2	Terminated early for lack of superiority vs placebo
SIK2/SIK3 inhibitor	GLPG3970	Oral dual SIK2/3 inhibitor	NCT04577794 (SEA TURTLE)	Phase 1b/2a	Completed 2021; no significant difference in Mayo Score reduction
Anti-CD3	Muronomab-CD3 (OKT3)	Murine anti-human IgG2a monoclonal antibody targeting CD3	NCT01287195	Phase 1b/2a	Terminated early when manufacturer discontinued OKT3 production
Combination	PF-06687234 + Infliximab	IL-10 fusion protein + TNF antagonist	NCT03269695	Phase 2a	Terminated Jan 2021 due to changed R&D strategy
Combination	SPY001, SPY002, SPY003	Anti-α4β7 + Anti-TL1A + Anti-IL-23 (various combinations)	NCT07012395	Phase 2	Started May 2025; estimated completion March 2028
Combination	Mirikizumab + Tirzepatide	IL-23p19 antibody + dual GLP-1/GIP receptor agonist	NCT06937086	Phase 3b	Started June 2025; estimated completion April 2028
Combination	Mirikizumab + Eltrekibart	IL-23p19 antibody + ELR+ CXC chemokine neutralizer	NCT06598943	Phase 2	Started Oct 2024; estimated completion Sept 2028
Combination	Guselkumab + Golimumab	IL-23p19 antibody + TNF antagonist	NCT03662542 (VEGA), NCT05242484 (DUET-UC)	Phase 2, Phase 2b	VEGA completed Nov 2021; 83% response with combination. DUET-UC primary completion May 2025
Combination	OD-07656 + Vedolizumab	IRAK4 inhibitor + α4β7 integrin antagonist	NCT06850727	Phase 2a	Started June 2025; estimated completion Nov 2026
Combination	Infliximab + Ustekinumab	TNF antagonist + IL-12/23 p40 antibody	NCT06453317	Phase 2	Started Feb 2025; estimated completion June 2028
Combination	AMT-101 + Adalimumab	IL-10 fusion protein + TNF antagonist	NCT04583358 (LOMBARD), NCT05372939 (MARKET)	Phase 2a	Both completed by July 2022; LOMBARD failed primary endpoint

## 2 Emerging therapeutic drugs in phase II and III trials

**Figure 3:**
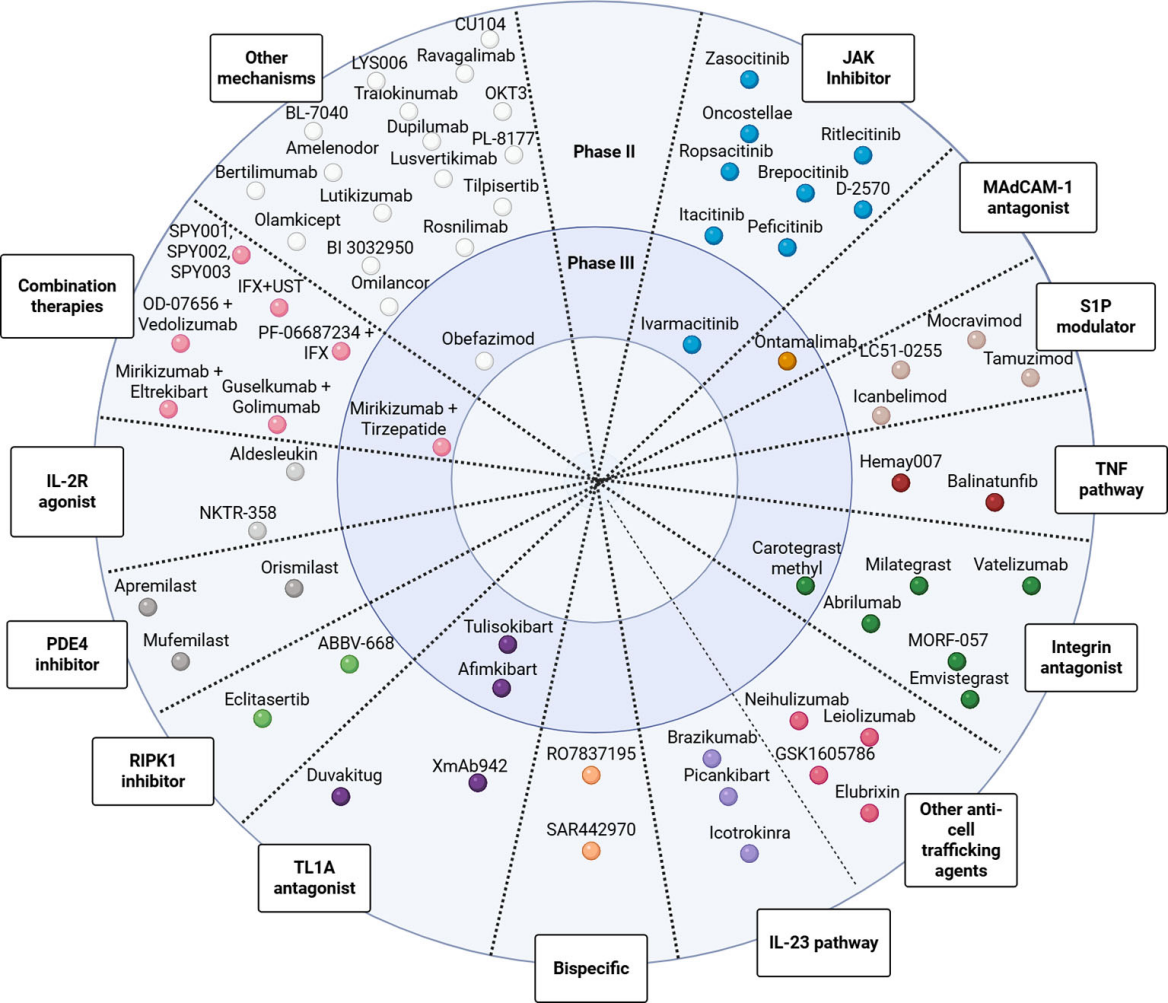
Therapeutic agents in development for ulcerative colitis by mechanism of action and clinical phase. Created with Biorender.com.

**Figure 3** illustrates the spectrum of investigational therapies for UC, organized by mechanism of action and stage of clinical development.

**Figure 4:**
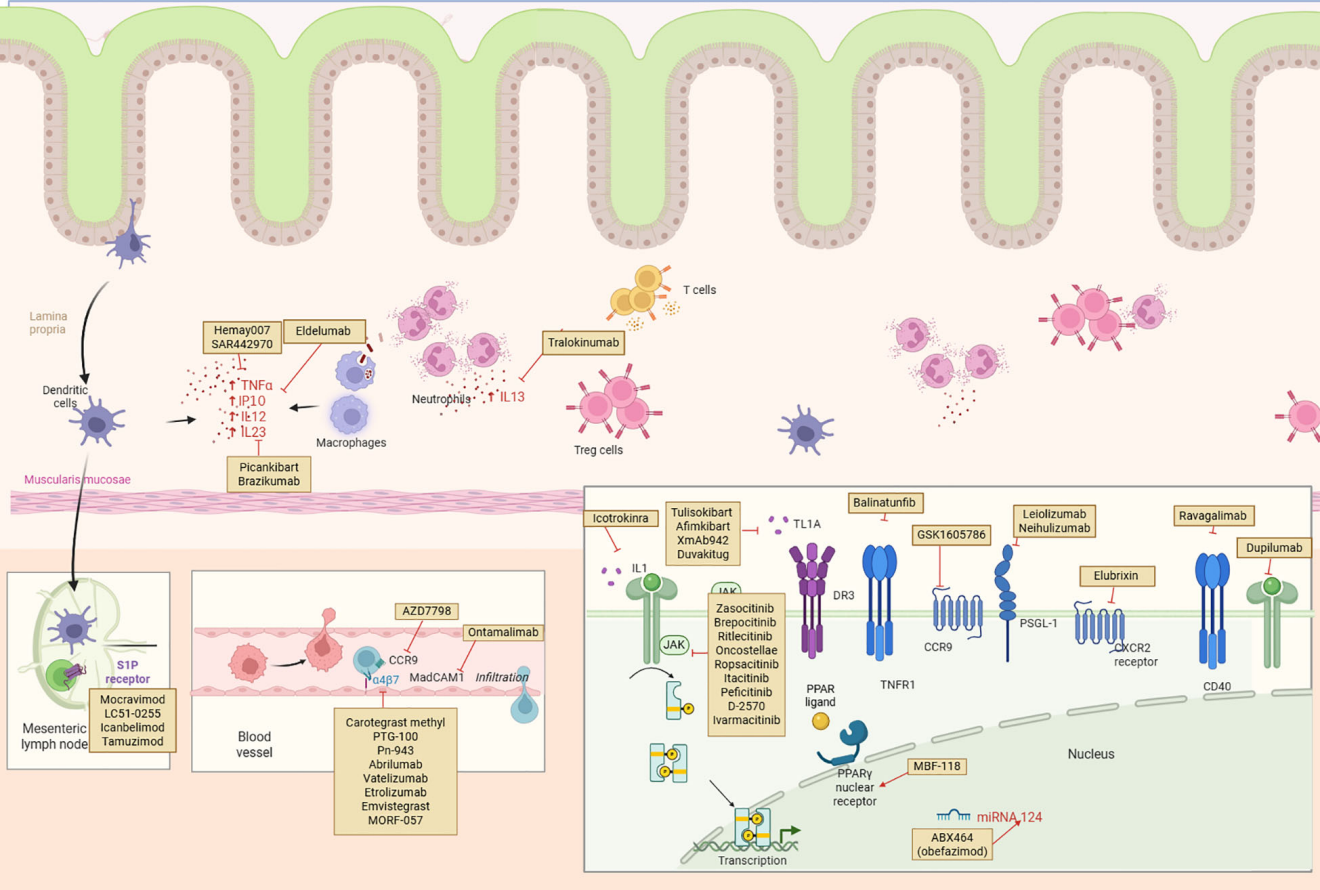
Mechanistic targets of emerging therapies in the ulcerative colitis pipeline. Created with Biorender.com.

**Figure 4** illustrates the diverse therapeutic targets of novel agents currently under investigation for ulcerative colitis, depicting their sites of action across the intestinal mucosa, vasculature, lymphoid tissue, and intracellular signaling pathways involved in inflammation and immune cell trafficking.

### 2.1 JAK inhibitors

Recent research has increasingly concentrated on the JAK-STAT signaling cascade, yielding innovative therapeutic approaches for improving IBD management (31, 32). The JAK family comprises four intracellular tyrosine kinases: JAK1, JAK2, JAK3, and TYK2, all of which are essential for relaying cytokine-mediated signals through the STAT pathway. Cytokine receptors trigger the activation of these kinases, promoting inflammation by driving T-cell proliferation and differentiation alongside B-cell activation. Within IBD pathophysiology, IL-6, IL-12, and IL-23 function as primary mediators of disease progression, exerting their effects through the Janus Kinase-Signal Transducer and Activator of Transcription (JAK-STAT) signaling axis. Interrupting this pathway’s activation effectively suppresses multiple inflammatory chemokines that contribute to disease pathology. Three JAK-inhibitors have been approved for the treatment of UC. Tofacitinib demonstrated clinical remission in 18.5% and 16.6% of patients at 8 weeks during induction trials, compared to 8.2% and 3.6% in placebo groups (22). For maintenance at 52 weeks, remission rates were 34.3% with 5mg twice daily and 40.6% with 10mg twice daily, versus 11.1% for placebo. Tofacitinib has shown promise in acute severe UC, with 83% treatment response compared to 59% with placebo, and reduced short-term colectomy risk (33). Filgotinib achieved clinical remission in 26.1% of biologic-naïve patients and 11.5% of biologic-experienced patients at 10 weeks, compared to 15.3% and 4.2% placebo rates respectively (26). Maintenance remission at 58 weeks was 37.2% versus 11.2% for placebo. Upadacitinib showed clinical remission rates of 26% and 33% at 8 weeks versus 5% and 7% for placebo (25). Maintenance remission at 52 weeks was 42% with 15mg daily and 52% with 30mg daily, compared to 12% for placebo. Meta-analyses consistently ranked upadacitinib as the most efficacious treatment for inducing and maintaining endoscopic, histological and clinical remission (34).

Currently, several pan-JAK inhibitors and selective JAK inhibitors, including novel TYK2 inhibitors, underwent recent or on-going development at different Phases for IBD treatment.

#### 2.1.1 Ivarmacitinib

Ivarmacitinib (SHR0302) is a selective JAK1 inhibitor designed to block the JAK-signal transducer and activator of transcription pathway, thereby controlling inflammation in UC. The Phase 2 trial (NCT03675477) employed a randomized, double-blind, placebo-controlled, multicenter design with 164 participants testing three different dosing regimens (4mg daily, 4mg twice daily, and 8mg daily) against placebo over an initial 8-week period, followed by an optional 8-week extension Phase where placebo patients were re-randomized to active treatment arms. The study included patients aged 18–75 with moderate to severe active UC, defined with 5–9 of the modified Mayo score (mMS), and with a Mayo endoscopic subscore (MES) ≥ 2. Patients needed to have had at least three months of disease history, while excluding those with indeterminate colitis, proctitis-limited disease, or treatment-naïve patients. The study completed in February 2021 and demonstrated statistically significant improvements in clinical response rates at week 8 for all ivarmacitinib doses (43.9% to 46.3%) compared to placebo (26.8%), with clinical remission rates of 22.0% to 24.4% versus 4.9% for placebo, while maintaining a favorable safety profile with no deaths, major cardiovascular events, or thromboembolic complications reported during the 18-week study period (35).

The study led to the current Phase 3 trial (NCT05181137)) which began in November 2021 and, based on its estimated completion date, is anticipated to be ongoing; however, no interim results have been publicly reported to date.

#### 2.1.2 Itacitinib

Itacitinib (INCB039110) is a JAK1 inhibitor developed for treating moderate to severe UC. The Phase 2 trial (NCT03627052) was designed as a double-blind, dose-ranging, placebo-controlled RCT with an open-label extension, planned to evaluate multiple oral dosing regimens of itacitinib administered once or twice daily compared to placebo over an 8-week primary treatment period. The study targeted patients aged 18–74 with confirmed UC for at least 12 weeks, requiring a 3-component mMS of 4–9 with MES ≥ 2, rectal bleeding subscore (RBS) ≥ 1, and stool frequency subscore (SFS) ≥ 1, plus failure or intolerance to at least one standard therapy including corticosteroids, immunosuppressants, or biologics like infliximab or vedolizumab.

The primary outcome measured clinical response at week 8, while secondary endpoints included endoscopic response, mucosal healing, endoscopic and clinical remission rates, changes in MS, quality of life measures, pharmacokinetic parameters, and safety assessments over approximately 60 weeks. However, the study was withdrawn in November 2019 due to lack of patient recruitment with zero participants enrolled and no safety concerns reported.

#### 2.1.3 Peficitinib

Peficitinib is an oral pan-JAK inhibitor that was studied in a Phase 2b dose-ranging RCT (NCT01959282) evaluating four dosing regimens (25 mg once daily, 75 mg once daily, 150 mg once daily, and 75 mg twice daily) versus placebo in 219 patients with moderate-to-severe UC (36). The primary outcome measured peficitinib dose-response at Week 8 using MS change from baseline, with secondary endpoints including clinical response, clinical remission, mucosal healing, IBDQ improvement, and inflammatory biomarker normalization at Week 8, plus treatment responses through Weeks 16 and 32. Inclusion criteria specified patients with moderate-to-severe UC, and the study included safety assessment through Week 36 or 4 weeks after the last dose, with results showing no statistically significant dose-response at Week 8, though numerically greater proportions of patients receiving doses ≥ 75 mg once daily achieved clinical outcomes, while treatment-emergent AEs were more frequent in peficitinib groups, particularly at higher doses (36). However, the UC development program for peficitinib was subsequently discontinued and did not advance to Phase 3 trials. While the company did not publicly disclose specific reasons for discontinuation, the lack of statistically significant dose-response at Week 8 in the Phase 2b trial, likely contributed to the decision not to pursue further development in UC.

#### 2.1.4 Oncostellae

Oncostellae (OST-122) is an oral, gut-restricted JAK3/TYK2/5′ adenosine monophosphate–activated protein kinase–related kinase 5 (ARK5) inhibitor designed for localized treatment of IBD with minimal systemic absorption to reduce toxicity risks associated with other JAK inhibitors. The Phase Ib/IIa randomized, double-blind, placebo-controlled multicenter RCT (NCT04353791) enrolled 32 patients with moderate to severe UC across 16 locations in Spain and Ukraine, randomizing 24 subjects to receive Oncostellae and 8 to placebo for 28 days. Inclusion criteria required patients aged 18–75 years with established UC for at least 3 months, inadequate response or intolerance to conventional treatments, MES ≥2, total MS 5-10, and stable concurrent medications, while excluding those with fulminant colitis, recent use of various immunosuppressive agents, active infections, or significant comorbidities.

The study completed in December 2022 and results demonstrated that OST-122 was well-tolerated with no SAEs or significant safety concerns, achieving minimal plasma concentrations (average Cmax 0.92 ng/mL on day 28) while maintaining therapeutically relevant colon tissue levels (15.5 μg/g tissue), confirming the compound’s gut-restricted profile (37). Clinical efficacy showed promising trends with 63% of OST-122 patients experiencing improved rectal bleeding versus 33% on placebo, 44% achieving clinical response versus 11% on placebo, and 22% reaching clinical remission, though statistical significance was not achieved due to the small study size and patient variability.

Thanks to the positive safety signals and promising trends in clinical efficacy in this Phase 1b/2a trial, the development program for Oncostellae in UC is continuing in development as a gut-restricted JAK inhibitor approach.

#### 2.1.5 Ritlecitinib

Ritlecitinib is an oral selective JAK3/TEC family kinase inhibitor studied in the VIBRATO trial (NCT02958865), a Phase 2b double-blind placebo-controlled RCT umbrella study that evaluated its efficacy in moderate-to-severe UC patients aged 18–75 years with a total MS ≥6, RBS ≥1, and MES ≥2. The 8-week induction period demonstrated statistically significant dose-dependent reductions in total MS compared to placebo, with the primary endpoint showing placebo-adjusted mean reductions of -2.0, -3.9, and -4.6 points for the 20mg, 70mg, and 200mg doses respectively (all p<0.01), alongside higher rates of modified clinical remission, with AEs being mostly mild and including infections in 8.7% of ritlecitinib patients versus 4.0% in placebo (38).

The study completed in May 2021 with 319 enrolled patients, though ritlecitinib was subsequently prioritized for alopecia areata development rather than UC, receiving FDA approval for severe alopecia areata in June 2023.

#### 2.1.6 Brepocitinib

Brepocitinib (PF-06700841) is an oral dual TYK2/JAK1 inhibitor evaluated alongside ritlecitinib in the same VIBRATO Phase 2b trial (NCT02958865) for moderate-to-severe UC with identical inclusion criteria of patients aged 18–75 years having total MS ≥6, RBS ≥1, and MES ≥2 (38). The 8-week induction period showed statistically significant dose-dependent improvements in total MS versus placebo, with placebo-adjusted mean reductions of -1.8, -2.3, and -3.2 points for the 10mg, 30mg, and 60mg doses respectively (all p ≤ 0.009), along with higher rates of modified clinical remission, though brepocitinib showed a higher infection rate at 16.9% compared to 4.0% for placebo and experienced one serious thromboembolic event considered unrelated to study drug.

The study completed in May 2021 with the same 319 total enrolled patients, and like ritlecitinib, brepocitinib was also advanced to alopecia areata trials rather than continued UC development, demonstrating acceptable short-term safety profiles in both indications.

#### 2.1.7 Zasocitinib

Zasocitinib (TAK-279) is an AI-designed, orally active, highly selective allosteric TYK2 inhibitor that blocks IL-23, IL-12, and type I interferon (IFN) signaling pathways without affecting other JAK kinases, showing the single amino acid difference (Ile597 in JAK1 versus Ala671 in TYK2) that confers zasocitinib’s >1.3 million-fold selectivity for TYK2 over JAK1. The binding affinity data (Kd = 0.0038 nM for TYK2 versus 4975 nM for JAK1) demonstrates the exceptional selectivity. It is being tested in a Phase 2, multicenter, placebo-controlled, double-blind RCT for moderately to severely active UC in 207 participants aged 18–75 who have inadequate response to conventional, biologic, or advanced therapies, with the primary outcome measuring clinical remission at week 12 using mMS, currently recruiting with estimated completion in August 2027 (NCT06254950).

Additionally, a Phase 2 open-label extension study evaluates long-term safety and efficacy of zasocitinib in 183 participants aged 18–76 with both UC and CD who demonstrated clinical or symptomatic response at week 52 of their parent trials, focusing on treatment-emergent AEs and clinically significant changes in vital signs, laboratory values, and ECG over 108 weeks of continued treatment, having started in May 2025 with completion expected in December 2029 (NCT06764615).

#### 2.1.8 Ropsacitinib

Ropsacitinib (PF-06826647) is a selective TYK2 inhibitor that was evaluated in, a Phase 2B double-blind, placebo-controlled RCT with an open-label extension (NCT04209556) designed to assess its safety and efficacy in participants with moderate to severe UC. The study tested four different dosing regimens (100 mg, 300 mg, 600 mg daily, plus 400 mg daily in the extension Phase) against placebo, with primary outcomes measuring endoscopic response at 8 weeks and safety parameters at 60 weeks, while secondary outcomes included clinical remission, endoscopic remission, and mucosal healing. Participants aged 18–75 years with moderate to severe UC (MS ≥6, RBS ≥1, MES ≥2) who had inadequate response to conventional therapies including corticosteroids, immunosuppressants, TNFα antagonists, anti-integrin inhibitors, JAK inhibitors, or anti-IL-12/IL-23 inhibitors were eligible for inclusion. The study was withdrawn following strategic portfolio re-prioritization by Pfizer with zero actual enrollment, meaning no participants were recruited and no results are available despite the planned completion date of October 2023.

#### 2.1.9 D-2570

D-2570 is a novel oral TYK2 inhibitor being developed for autoimmune diseases including psoriasis and UC evaluated in a Phase 2 multicenter double-blind, placebo-controlled RCT (NCT07035041) with 120 participants randomized 1:1:1 to two different D-2570 dosing arms or placebo for 12 weeks. The primary outcome measures the proportion of subjects achieving clinical remission at Week 12 defined by mMS criteria including SFS ≤1 with ≥1-point decrease from baseline, RBS of 0, and MES ≤1, with inclusion criteria requiring established UC diagnosis ≥3 months duration, intestinal involvement ≥15 cm from anal verge, mMS 5–9 points, and inadequate response to standard treatments. The study began May 2025 with estimated primary completion July 2026 and final completion December 2026, currently in the recruiting Phase with no results yet available as the trial is ongoing.

### 2.2 Cell trafficking and adhesion

The controlled trafficking of immune cells to the intestinal mucosa represents a fundamental process in maintaining gut homeostasis and immunosurveillance (39). In IBD, this tightly regulated system becomes dysregulated, leading to excessive recruitment and accumulation of pathogenic immune cells that perpetuate chronic inflammation. The multi-step process of leukocyte migration involves sequential interactions between selectins, integrins, chemokine receptors, and their corresponding ligands expressed on endothelial cells and within intestinal tissues.

In healthy individuals, naïve lymphocytes traffic to gut-associated lymphoid tissue via interactions between α4β7 integrin and mucosal addressin cell adhesion molecule-1 (MAdCAM-1), supported by chemokine receptor signaling including CCR7 and CCR9. Following antigen encounter and activation in inductive sites, these primed lymphocytes acquire gut-homing properties and traffic to the lamina propria through similar integrin-MAdCAM-1 interactions. Tissue retention is further mediated by αEβ7 integrin binding to E-cadherin on epithelial cells, which maintains intraepithelial lymphocyte populations. Egress from lymphoid tissues is regulated by sphingosine-1-phosphate gradients between tissues and circulation.

In UC, this physiologic trafficking becomes pathologically amplified (40). Pro-inflammatory cytokines including TNF-α upregulate MAdCAM-1 expression on intestinal venules while inducing expression of additional adhesion molecules normally absent from the gut, including VCAM-1, ICAM-1, and peripheral lymph node addressin. Enhanced selectin expression and PSGL-1-mediated interactions promote increased leukocyte rolling on inflamed endothelium. Elevated chemokine signaling through CCL2, CCL20, CCL25, and CXCL10 drives recruitment of effector T cells, particularly Th1 and Th17 subsets that secrete pro-inflammatory cytokines perpetuating mucosal damage. Simultaneously, neutrophil recruitment via CXCR2 signaling in response to elevated IL-8 and related chemokines contributes to acute inflammatory responses and tissue damage. This creates a self-amplifying cycle where recruited immune cells produce mediators that enhance further trafficking and activation.

Sphingosine-1-phosphate (S1P) is a bioactive phospholipid that plays a critical regulatory role in lymphocyte trafficking, immune cell activation, and inflammatory processes (41). S1P exerts its effects through binding to five G-protein coupled receptors (S1PR1-5) with variable tissue distribution: S1PR1–3 are ubiquitously expressed, S1PR4 is predominantly located in lymphoid tissue and lungs, while S1PR5 is primarily expressed in the central nervous system and spleen. Within the immune system, S1P regulates the egress of lymphocytes, particularly B and T cells, from lymph nodes into blood and lymph through concentration gradients. Naïve CD4+ and CD8+ T cells activated by antigen-presenting dendritic cells within lymph nodes migrate along increasing S1P gradients from lymphoid tissues (low S1P) to circulation (high S1P) via S1PR1 signaling. In IBD, S1P has been found to be overexpressed in experimental colitis models and in patients with active UC, making S1P receptor modulation an attractive therapeutic target (42). S1P receptor modulators act as functional antagonists despite being receptor agonists. Upon binding to S1PR1 on lymphocyte surfaces, these agents cause persistent receptor internalization and proteasome-mediated degradation, resulting in functional downregulation (43). The consequent absence or reduction of surface S1PR1 renders lymphocytes unable to sense the S1P gradient, effectively sequestering them within lymph nodes and secondary lymphoid organs.

Anti-trafficking therapies aim to interrupt this pathologic cascade through several complementary mechanisms. Integrin antagonists block the interaction between α4β7 and MAdCAM-1, preventing lymphocyte adhesion to and transmigration across intestinal endothelium. MAdCAM-1 antagonists target the endothelial ligand rather than the leukocyte receptor. PSGL-1 antagonists disrupt the initial selectin-mediated rolling and tethering of leukocytes on activated endothelium, representing an upstream intervention in the trafficking cascade. Chemokines orchestrate directional migration of immune cells through concentration gradients, with multiple chemokine pathways contributing to pathogenic leukocyte recruitment in UC, including CXCL10-CXCR3 axis for T cell trafficking, CXCL8-CXCR2 axis for neutrophil recruitment, and CCL11 for eosinophil migration. CXCR2 antagonists block neutrophil recruitment and activation driven by IL-8 and related chemokines, addressing the innate immune component of intestinal inflammation. S1PR modulators sequester lymphocytes within lymphoid organs by preventing their egress into circulation.

Vedolizumab, a humanized IgG1 monoclonal antibody that specifically targets the α4β7 integrin heterodimer without affecting α4β1, represents the prototypical gut-selective anti-trafficking agent approved by FDA, EMA, and PMDA based on the GEMINI trials demonstrating superior efficacy over placebo for both induction and maintenance in moderate to severe UC (GEMINI 1: 47.1% clinical response and 16.9% remission at week 6; 41.8% remission at week 52) with the VARSITY head-to-head trial showing superiority over adalimumab for clinical remission in UC (31.3% vs 22.5% at week 52, p=0.006) (18, 44). Vedolizumab’s mechanism involves selective inhibition of α4β7-MAdCAM-1 binding to prevent gut-homing of lymphocytes while preserving systemic immunity as demonstrated by maintained humoral responses to parenteral vaccines but blocked responses to oral vaccines, with proposed effects on naïve T and B cell trafficking to intestinal inductive sites and attenuation of lymphoid aggregates contributing to therapeutic benefit beyond simple effector cell blockade.

Ozanimod, a selective S1PR1/5 modulator requiring dose escalation (0.23mg days 1-4, 0.46mg days 5-7, then 0.92mg daily), received FDA and EMA approval in 2021 based on the True North program demonstrating 18.4% clinical remission at week 10 induction and 37.0% at week 52 maintenance versus 6.0% and 18.5% placebo respectively, with favorable safety profiles though requiring cardiovascular monitoring and dose titration to mitigate first-dose heart rate effects (24, 45). Etrasimod, a more selective S1PR1/4/5 modulator requiring no dose escalation and administered at 2mg once daily from initiation, achieved FDA approval in 2023 and EMA approval in 2024 based on ELEVATE UC 52 and UC 12 demonstrating 27% clinical remission at week 12 and 32% at week 52 versus 7% placebo at both timepoints, with rapid symptomatic improvement by day 2 and maintained efficacy in biologic/JAK-experienced patients (17-21% remission) and notably in isolated proctitis (43% remission at week 12) (23).

This section reviews investigational anti-trafficking and adhesion molecules currently in Phase II and III clinical development for moderate to severe UC.

#### 2.2.1 Carotegrast methyl

Carotegrast methyl (AJM300) is an oral small-molecule α4-integrin antagonist tested in this multicenter, double-blind, placebo-controlled Phase III RCT (NCT03531892) involving 203 patients with moderately active UC who received either 960mg three times daily or placebo for 8 weeks (46). The primary outcome was clinical response rate at week 8, defined as ≥30% and ≥3-point reduction in MS, ≥1-point reduction in RBS ≤1, and MES ≤1, with inclusion criteria requiring MS 6-10, MES ≥2, RBS ≥1, and inadequate response/intolerance to mesalamine. The completed study demonstrated significant efficacy with 45% clinical response in the AJM300 group versus 21% in placebo (OR 3.30, 95% CI 1.73-6.29, p=0.00028), good tolerability with similar adverse event rates between groups, and established AJM300 as a potential novel induction therapy for moderately active UC.

#### 2.2.2 Milategrast

Milategrast (E6007) is a small molecule integrin antagonist that blocks the interaction between calreticulin and integrins on leukocytes to prevent immune cell adhesion and infiltration into the intestinal tissue. The Phase 2 double-blind, placebo-controlled RCT (NCT03018054) tested 30mg and 60mg doses versus placebo once daily for 8 weeks in 147 Japanese participants aged 20–74 with moderate active UC (MS 6-10, MES ≥2, RBS ≥1) who had inadequate response to standard treatments. The study was completed in August 2019 measuring change in MS from baseline at 8 weeks as the primary outcome, but development was discontinued in May 2022 due to business priorities despite the novel mechanism showing anti-inflammatory effects in preclinical IBD models.

#### 2.2.3 Abrilumab

Abrilumab, a human monoclonal IgG2 antibody targeting α4β7 integrin, was evaluated in two Phase 2 studies for moderate-to-severe UC. The global Phase 2b trial (NCT01694485) enrolled 354 patients aged 18–65 with Mayo Score 6–12 who had inadequate response or intolerance to conventional therapies, randomizing them to subcutaneous abrilumab at 7, 21, or 70 mg (day 1, weeks 2, 4, then every 4 weeks) or a single 210 mg dose versus placebo, with the primary endpoint of clinical remission at week 8 (Mayo Score ≤2, no subscore >1). Results showed significantly higher remission rates with abrilumab 70 mg (13.3%, OR 3.35, p=0.021) and 210 mg (12.7%, OR 3.33, p=0.030) versus placebo (4.3%), along with superior response rates (49.0% and 46.8% vs 25.9%) and mucosal healing rates (32.7% and 29.1% vs 21.6%), with good tolerability and no cases of progressive multifocal leukoencephalopathy or deaths (47).

A parallel Japanese Phase 2 study (NCT01959165) enrolled 44 patients with similar inclusion criteria, randomizing them to abrilumab 21, 70, or 210 mg versus placebo. While no formal statistical testing was performed due to the small sample size, the study demonstrated numerically higher remission rates at week 8 in the abrilumab groups overall (12.9%) compared to placebo (0%), with individual dose results of 10.0% (21 mg), 16.7% (70 mg), and 11.1% (210 mg), alongside numerically higher response rates with 210 mg (66.7%) and mucosal healing rates with 70 mg and 210 mg (33.3% and 44.4% respectively), confirming the favorable safety profile observed in the global study with no progressive multifocal leukoencephalopathy or deaths reported (48).

#### 2.2.4 Vatelizumab

Vatelizumab (SAR339658), a monoclonal antibody targeting α4β7 integrin, was evaluated in a double-blind, placebo-controlled Phase 2 RCT (NCT01659138) involving patients aged 18–70 with moderate to severe UC who had inadequate response to immunosuppressants or TNFα antagonists, confirmed by MS 6–12 and MES ≥2 with fecal calprotectin ≥200mg/kg. The primary outcome measured clinical response by MS at Week 8, with secondary outcomes including clinical remission, mucosal healing, quality of life measures, and safety assessments through Week 17.

A single-arm, open-label Phase 2 extension study (NCT01861249) evaluating the long-term safety and tolerability of SAR339658 over 62 weeks in patients with UC who had previously completed the initial 8-week treatment in the primary study (ACT12688) with acceptable safety profiles. Participants received SAR339658 every 2 or 4 weeks based on their clinical response, with the primary outcome measuring AEs up to Week 68 and secondary outcomes including clinical remission by MS, mucosal healing at Week 62, and changes in partial MS and quality of life measures, ultimately enrolling only 6 patients before completing in April 2016.

Both studies investigated the same investigational integrin antagonist for UC treatment but were terminated early due to recruitment challenges, limiting the ability to fully assess the therapeutic potential of this novel mechanism of action in inflammatory bowel disease.

#### 2.2.5 Emvistegrast

Emvistegrast is an oral α4β7 integrin inhibitor designed to block immune cell migration to the gastrointestinal tract, reducing intestinal inflammation in UC patients. A Phase 2 double-blind, placebo-controlled, multicenter RCT evaluates three different doses of GS-1427 against placebo over 76 weeks, with the primary endpoint measuring clinical response at week 12 defined as a decrease in mMS of at least 2 points with 30% reduction from baseline and improved RBS (NCT06290934). Participants must be adults aged 18–75 with moderately to severely active UC confirmed by endoscopy and histology, having at least 15 cm disease extent from anal verge, modified MS of 5–9 points with MES of at least 2, and inadequate response or intolerance to prior treatments including corticosteroids, immunomodulators, or advanced therapies, while excluding those with CD, toxic megacolon, prior vedolizumab exposure, or contraindicated medications, with the study completing primary outcomes in October 2025 and final completion estimated for February 2027.

#### 2.2.6 MORF-057

MORF-057 is an oral small-molecule inhibitor of α4β7 integrin that blocks lymphocyte recruitment to the gut and was evaluated in the EMERALD-1 study (NCT05291689), an open-label Phase 2a single-arm multicenter trial where 35 participants with moderately to severely active UC received 100 mg twice daily for 52 weeks following a 6-week screening period. Participants were aged 18–85 years with symptoms for at least 3 months, a modified MS of 5-9, MES of at least 2, and Robarts Histopathology Index score of at least 10, and the primary outcome was change in histopathology score from baseline to week 12, which showed a mean reduction of 6.4 points with 22.9% achieving remission at 12 weeks and 31.4% at 52 weeks. The study was completed on November 12, 2023, demonstrating that MORF-057 was well tolerated with no serious adverse events and promising efficacy, with mean histopathology score improvements of 13.5 points from baseline at week 52 in the 18 participants who completed treatment (49).

The same MORF-057 molecule is being evaluated in a double-blind, Phase 2b placebo-controlled RCT (EMERALD-2, NCT05611671) with 282 participants meeting similar inclusion criteria but specifically requiring inadequate response to aminosalicylates, corticosteroids, immunosuppressants, or advanced UC therapies, measuring clinical remission using mMS at 12 weeks as primary endpoint. The study design included three active dose regimens plus placebo during a 12-week induction period followed by maintenance dosing, with primary completion in November 2024 and estimated final completion in August 2026.

#### 2.2.7 Ontamalimab

Ontamalimab is a fully human IgG2 monoclonal antibody that selectively binds MAdCAM-1 to inhibit α4β7+ lymphocyte adhesion, tested in Phase III induction studies for moderate to severe UC, after Phase II studies (TURANDOT; NCT01620255; TURANDOT II, NCT01771809) (50). The first induction study FIGARO UC 301 (NCT03259334) randomized 380 participants in a 2:2:1 ratio to receive ontamalimab 25mg, 75mg, or placebo subcutaneously at weeks 0, 4, and 8, with the primary endpoint of clinical remission at week 12 showing the 75mg dose achieved statistically significant results at 29.8% versus 15.8% placebo while the 25mg dose did not reach significance at 18.5%, along with superior secondary endpoints including endoscopic response, clinical response, and mucosal healing all favoring the 75mg dose (51). The second induction study FIGARO UC 302 (NCT03259334) enrolled 279 participants using an identical design and demonstrated efficacy for both doses, with 25mg achieving clinical remission in 27.0% versus 12.5% placebo and 75mg achieving 29.5% versus 12.5% placebo, while both doses showed significant benefits across secondary endpoints including endoscopic response, clinical response, and mucosal healing, with inclusion criteria requiring patients aged 18–66 years with moderate to severe disease and the studies showing good tolerability with most common adverse events being UC worsening, arthralgia, and nasopharyngitis.

The maintenance study FIGARO UC 303 (NCT03290781) enrolled 366 responders who continued their induction dose or switched to placebo for 52 weeks, demonstrating that both 25mg and 75mg doses significantly outperformed placebo with clinical remission rates of 53.5% and 40.2% respectively versus approximately 8-13% placebo, while inclusion criteria required patients aged 18–66 years with moderate to severe disease and the completed studies showed favorable safety profiles with most common adverse events being UC worsening, arthralgia, and nasopharyngitis (51).

A meta-analysis combining all three studies confirmed ontamalimab’s superiority over placebo in UC patients across multiple outcomes: clinical remission (RR = 2.17, 95% CI 1.4≥2.32, p<0.01), clinical response (RR = 1.79, 95% CI 1.35-2.38, p<0.01), endoscopic response (RR = 2.27, 95% CI 1.55-3.31, p<0.01), and mucosal healing (RR = 2.39, 95% CI 1.63-3.50, p<0.01) (52). Safety profiles were comparable between ontamalimab and placebo groups across all studies, with no significant differences in AEs, SAEs, or study discontinuations due to AEs.

Despite positive Phase 3 results demonstrating a favorable benefit-risk profile, ontamalimab was not submitted for regulatory approval. Following Takeda’s acquisition of Shire, development was discontinued due to inability to find a licensee for the compound, rather than efficacy or safety concerns. Clinical trial data and biomaterials were subsequently donated to the Crohn’s and Colitis Foundation.

#### 2.2.8 LC51-0255

LC51–0255 is an investigational oral drug tested in this Phase 2, multicenter, placebo-controlled parallel group RCT (NCT04096573) evaluating clinical efficacy and safety in subjects with moderately to severely active UC (53). The study planned to randomize participants aged 18–80 years with endoscopically confirmed active UC and moderate to severe disease based on MS, while excluding those with severe extensive colitis, microscopic/ischemic/infectious colitis, or recent treatment with immunosuppressants or biologics within specified timeframes. The primary outcome was clinical remission at week 12 assessed by Mayo component sub-scores, with secondary outcomes including clinical response and endoscopic improvement, but the study was withdrawn due to company decision with zero actual enrollment and no results available.

#### 2.2.9 Mocravimod

This Phase 2 randomized, double-blind, placebo-controlled study (NCT01375179) evaluated Mocravimod (KRP203), an oral S1PR modulator, in 27 patients with moderately active refractory UC who had inadequately responded to or were intolerant of 5-ASA therapy. The primary outcome was clinical remission rate at 8 weeks, with secondary outcomes including safety, tolerability, pharmacokinetics, and inflammatory markers over 12 weeks of follow-up. The study was terminated early in May 2012 after enrolling only 27 of the planned 72 participants, suggesting the trial did not demonstrate sufficient efficacy to warrant continuation.

#### 2.2.10 Tamuzimod

Tamuzimod (VTX002) is a selective sphingosine 1-phosphate receptor 1 modulator studied in a Phase 2, multicenter, double-blind, randomized, placebo-controlled trial (NCT05156125) involving 213 patients with moderately-to-severely active UC who had inadequate response or intolerance to conventional or advanced therapies, randomized 1:1:1 to receive once-daily oral tamuzimod 60mg, 30mg, or placebo for 13 weeks (54). The primary endpoint was clinical remission at week 13, defined as mMS SFS ≤1, RBS of 0, and MES ≤1, which was achieved by 28% of patients receiving tamuzimod 60mg versus 11% receiving placebo (p=0.018), with secondary endpoints including endoscopic improvement, symptomatic remission, and histologic remission all showing nominal statistical significance. The study completed in August 2023 and demonstrated that tamuzimod was well-tolerated with most AEs being mild to moderate, including upper respiratory tract infections, anemia, and headache, while showing no cases of bradycardia, atrioventricular block, macular edema, severe infections, malignancies, or deaths, supporting continued Phase 3 development for UC treatment.

#### 2.2.11 Elubrixin

CXCR2 is a chemokine receptor expressed on neutrophils that responds to IL-8 (CXCL8) and related CXC chemokines, mediating neutrophil recruitment and activation in acute inflammatory responses characteristic of UC flares. Elubrixin (SB-656933) is an orally active selective CXCR2 antagonist and IL-8 receptor antagonist that was investigated in a Phase 2, open-label, 7-day repeat dose study (NCT00748410) to evaluate its pharmacodynamics in patients with UC using 99mTc-HMPAO leukocyte SPECT scintigraphy to measure neutrophil migration to inflamed tissue. The study included patients aged 18–65 with moderately active UC (MES ≥2) for at least 3 months, requiring normal liver enzymes, effective contraception, and excluding those with mild disease, recent drug use, infections, or significant comorbidities. The study was terminated early after enrolling only 3 participants out of a planned larger cohort, completing in December 2009, making it impossible to draw meaningful conclusions about the compound’s efficacy in reducing neutrophil accumulation in the colon, though the limited safety data showed the drug was well-tolerated with no specific AEs reported.

#### 2.2.12 Leiolizumab

PSGL-1 (P-selectin glycoprotein ligand-1, CD162) mediates the initial capture and rolling of leukocytes on activated endothelium through interactions with P-selectin and E-selectin, representing an early step in the trafficking cascade upstream of integrin-mediated firm adhesion. Leiolizumab is a tetravalent agonist IgG1 antibody targeting PSGL-1 being evaluated in a Phase 2a, multicenter, single-arm, open-label study (NCT06109441) for moderately to severely active UC refractory to biologic therapy. The study enrolls approximately 50 participants aged 18–75 with UC confirmed for at least 12 weeks, a mMS of 5–9 with MES ≥2 and RBS ≥1, involving at least 15 cm of colon, and previous inadequate response to 1–2 advanced therapies, while excluding those with other forms of colitis, recent biologic use within 56 days, or significant comorbidities. The primary outcome measures change from baseline in mMS at week 12, with secondary endpoints including clinical response and remission rates, endoscopic improvement, histological remission using both Robarts Histopathology Index and Geboes scoring systems, and corticosteroid-free remission rates assessed at weeks 12 and 52, with the study having started in December 2023 and estimated completion by November 2026, though no results are currently available.

#### 2.2.13 Neihulizumab

Neihulizumab (ALTB-168) is an PSGL-1 IgG4 monoclonal antibody that acts as an immune checkpoint agonist by downregulating activated T-cells in UC treatment. a Phase II open-label, single-arm, multicenter trial. (NCT03298022) tested two dosing regimens of intravenous neihulizumab in 24 patients with moderate to severe active UC who were refractory or intolerant to TNFα antagonists and/or anti-integrin therapies, with the primary endpoint being clinical response defined as ≥3-point MS reduction at week 12. The study was terminated due to COVID-19 operational difficulties but completed with actual enrolment of 24 patients from May 2018 to June 2020, showing clinical response rates of 22% in the 5 + 3 regimen and 50% in the 8 + 2 regimen at week 12, with generally acceptable safety profiles and 93% of patients reporting AEs mostly unrelated to treatment (55).

#### 2.2.14 GSK1605786

CCR9 and its ligand CCL25 primarily mediate lymphocyte trafficking to the small intestine, and while this pathway was explored for UC treatment, clinical development was discontinued before enrollment. GSK1605786 is an oral CCR9 chemokine receptor antagonist (500 mg twice daily) that selectively blocks lymphocyte trafficking to the small bowel, developed for treating IBD. A Phase II double-blind, placebo-controlled RCT (NCT01658605) designed to evaluate efficacy and safety over 16 weeks in patients with active UC extending beyond the rectum with MS of 5-10, measuring ordinal response at week 12 as the primary outcome alongside safety parameters and biomarkers. However, this multi-center European study was terminated prior to enrolling any of the planned participants, with the pharmaceutical company deciding to delay pursuit of this indication as the underlying biology evolved, resulting in zero actual enrollment and no results available.

### 2.3 TNF-α pathway inhibitors

TNF-α is a pleiotropic pro-inflammatory cytokine that plays a central role in IBD pathogenesis, produced predominantly by activated macrophages, monocytes, and T lymphocytes within inflamed intestinal mucosa (56). TNF-α exerts its biological effects through two distinct receptors: TNFR1 (p55), ubiquitously expressed and mediating pro-inflammatory signaling, apoptosis, and necroptosis; and TNFR2 (p75), expressed primarily on immune and endothelial cells and mediating immunoregulatory functions, tissue repair, and regulatory T cell expansion (57).

Anti-TNF biologics revolutionized IBD treatment beginning in the late 1990s and early 2000s, establishing the paradigm of targeted biological therapy for moderate to severe disease. Three anti-TNF monoclonal antibodies are currently approved for UC: infliximab (chimeric mouse-human IgG1), adalimumab (fully human IgG1), and golimumab (fully human IgG1). These agents neutralize TNF-α through multiple mechanisms including blocking TNF-α binding to both receptors, inducing complement-dependent cytotoxicity and antibody-dependent cell-mediated cytotoxicity of TNF-expressing cells, and triggering reverse signaling through transmembrane TNF leading to immune cell apoptosis. Infliximab (5–10 mg/kg IV at weeks 0, 2, 6, then every 8 weeks) demonstrated 61-69% clinical response at week 8 versus 29-37% placebo in ACT 1/2 trials (p<0.001), with sustained response of 44-45% at week 54 versus 20% placebo and 22% achieving corticosteroid-free remission (17). Adalimumab (160mg/80mg/40mg every 2 weeks SC) achieved 16.5% remission at week 8 versus 9.3% placebo (p=0.019) and 17.3% at week 52 versus 8.5% placebo (p=0.004) in ULTRA 2, with superior efficacy in anti-TNF naïve patients (21.3% and 22% remission) compared to experienced patients (9.2% and 10.2%) (58). Golimumab (200mg/100mg then 50-100mg every 4 weeks SC) induced 51-55% clinical response at week 6 versus 30.3% placebo (p ≤ 0.0001) in PURSUIT trials (59).

Despite transformative impact, anti-TNF therapies have significant limitations driving next-generation development. Primary non-response occurs in 20-40% of patients due to TNF-independent pathways, inadequate drug concentrations, or mechanistic resistance. Secondary loss of response affects approximately 13% per year (cumulative 50% over time), predominantly caused by immunogenicity with anti-drug antibodies (ADAs) developing in 10-30% of infliximab-treated and 5-15% of adalimumab-treated patients, accelerating drug clearance and neutralizing activity. Infliximab’s chimeric structure containing murine sequences increases immunogenicity, often necessitating combination with immunomodulators (azathioprine) to suppress ADAs, though this increases infection and lymphoproliferative disorder risks (11). Pharmacokinetic variability related to body weight, disease burden, fecal drug loss, and Fc receptor polymorphisms contributes to suboptimal exposure, leading to dose escalation requirements in 30-50% of patients.

Next-generation TNF pathway inhibitors employ several innovative strategies to address these limitations: selective TNFR1 antagonists preserving TNFR2 signaling (balinatunfib/SAR441566), bispecific antibodies targeting TNF-α plus additional mediators for synergistic blockade (SAR442970 targeting TNF-α and OX40L), small molecule TNF modulators avoiding immunogenicity (hemay007), CD40 antagonists indirectly modulating TNF-related pathways (ravagalimab), enhanced imaging techniques using fluorescently labeled agents to optimize dosing (adalimumab-680LT), and biosimilar formulations providing increased access (CT-P13). These approaches aim to improve upon the 30-60% clinical response and 15-35% remission rates of current anti-TNF agents while reducing immunogenicity, optimizing pharmacokinetics, and enhancing safety profiles.

The following section reviews investigational TNF pathway inhibitors currently in Phase II and III clinical development for moderate to severe UC.

#### 2.3.1 Hemay007

A Phase II study (NCT03977480) evaluated Hemay007, a small molecule that acts as a TNF-α regulator, using a multicenter, randomized, double-blind, placebo-controlled design with four treatment arms comparing different dosing regimens (400 mg BID, 800 mg QD, 600 mg BID, and placebo) over 12 weeks followed by an open-label extension. The primary outcome measured clinical response defined as MS reduction of ≥3 points and ≥30% from baseline with RBS improvement, while inclusion criteria required patients aged 18–70 years with active UC (MS ≥4, MES ≥2) who had treatment failure or intolerance to standard therapies including 5-ASA, corticosteroids, immunomodulators, or anti-TNF agents. The study was terminated early due to recruitment difficulties, completing in August 2022 with only 70 of the planned 288 patients enrolled, and no efficacy or safety results have been posted on ClinicalTrials.gov.

#### 2.3.2 SAR442970

SAR442970 is a bispecific pentavalent Nanobody administered subcutaneously that simultaneously targets TNF-α and OX40L, containing two domains each binding to these targets plus one albumin-binding domain for extended half-life. A Phase 2b double-blind, placebo-controlled RCT (NCT06975722) uses a parallel assignment design with quadruple masking, testing two different SAR442970 dose regimens against placebo via subcutaneous administration. Participants must be adults aged 18–75 with moderate-to-severe UC for at least 3 months confirmed by endoscopy, mMS 5-9, and either inadequate response to standard treatments or advanced therapies, while excluding those with CD, recent infections, immunosuppression, or other inflammatory conditions. The study began July 2025 with primary completion estimated December 2026 and study completion October 2029, including 16-week induction, maintenance period, and optional long-term extension up to 104 weeks for eligible participants.

#### 2.3.3 Balinatunfib

Balinatunfib (SAR441566) is a small molecule TNF inhibitor that stabilizes an asymmetric form of soluble TNF, preventing TNFR1 signaling while sparing TNFR2 pathways (60). It is being evaluated in a Phase 2 multinational, multicenter, randomized, double-blind, placebo-controlled, dose-ranging study (NCT06867094) in adults aged 18–75 years with moderate-to-severe active UC (disease duration ≥3 months, mMS 5-9) who had inadequate response, loss of response, or intolerance to standard treatments or advanced therapies including biologics or small molecules. The primary outcome measures clinical remission at Week 12 defined by modified Mayo Score ≤2 with no subscore >1, while secondary outcomes include clinical response, endoscopic remission and improvement, histologic-endoscopic mucosal improvement, and quality of life measures using the IBDQ questionnaire, evaluated over a 52-week double-blind treatment period comprising 12 weeks induction and 40 weeks maintenance with optional 40-week open-label extension. The study began recruiting in March 2025 with estimated primary completion in July 2027 and final completion in May 2028, enrolling approximately 204 participants across 62 locations in multiple countries, with no results currently available as the trial is actively recruiting.

#### 2.3.4 Ravagalimab

Ravagalimab (ABBV-323) is a IgG1 monoclonal antibody that antagonizes CD40, a TNF receptor family member involved in lymphocyte activation and antigen-presenting cell function. A Phase 2a multicenter, single-arm, open-label study (NCT03695185) enrolled 42 participants with moderate to severe UC who had failed prior biologic therapies, administering ravagalimab 600 mg IV at week 0 followed by 300 mg subcutaneous doses at weeks 2, 4, 6, 8, and 10. The primary outcome measured the proportion of participants achieving endoscopic improvement (MES subscore of 0 or 1) at week 8, with secondary outcomes including clinical remission and response rates per various MS criteria, and the study was completed in September 2021 through specific efficacy and safety results are not available. The ravagalimab Phase 2 single-arm study evaluated this anti-CD40 monoclonal antibody in moderate to severe UC patients by constructing synthetic placebo control groups using two approaches: first, a Bayesian method incorporating historical placebo data from meta-analyses of tofacitinib, ustekinumab, and upadacitinib pivotal trials, taking advantage of objective centrally-read endoscopies and low placebo endoscopic improvement rates; and second, 1:1 propensity score matching using individual-level data from upadacitinib registration trials with greedy nearest-neighbor matching algorithms to balance covariates between treatment and control groups. This innovative approach allowed all patients to receive active treatment while still providing comparative efficacy data, demonstrating promising safety and efficacy results that supported continued development of ravagalimab as a potential UC therapeutic without the ethical and recruitment challenges of traditional placebo-controlled trials.

### 2.4 Anti interleukins and anti-interleukin receptors

Interleukins comprise a diverse family of cytokines orchestrating immune cell communication, differentiation, and effector functions, with multiple interleukin pathways contributing to chronic intestinal inflammation in UC. The IL-23/IL-12 axis has emerged as a central driver of IBD pathogenesis, with IL-23 (a heterodimeric cytokine composed of unique p19 and shared p40 subunits) promoting expansion and maintenance of pathogenic Th17 cells that produce IL-17A, IL-17F, IL-22, and GM-CSF, cytokines that damage intestinal epithelium, disrupt barrier function, and recruit neutrophils (61). Genome-wide association studies have identified multiple IBD susceptibility loci within IL-23 pathway genes (IL23R, IL12B, JAK2, TYK2, STAT3), providing strong genetic validation for therapeutic targeting. Beyond IL-23/IL-12, other interleukins contribute to UC pathogenesis: IL-6 drives acute Phase responses and Th17 differentiation through trans-signaling via soluble IL-6 receptor-gp130 complexes; IL-13 and IL-4 mediate Th2-driven epithelial dysfunction and tissue remodeling; IL-36 family members amplify neutrophilic inflammation; IL-1β activates innate immunity and enhances adaptive responses; IL-7 maintains pathogenic T cell survival; and IL-10 deficiency impairs regulatory mechanisms.

Currently approved anti-interleukin biologics targeting IL-23/IL-12 have demonstrated substantial efficacy in moderate to severe UC. Ustekinumab, a fully human IgG1 antibody targeting the shared p40 subunit of both IL-23 and IL-12, achieved 15.6% week 8 remission (6 mg/kg IV induction) versus 5.3% placebo and 43.8% week 44 remission (90mg SC every 8–12 weeks) versus 24.0% placebo in UNIFI trials (14). Selective IL-23p19 inhibitors, which preserve IL-12/Th1 responses while specifically blocking pathogenic Th17 pathways, demonstrate superior efficacy. Mirikizumab (300mg IV at weeks 0, 4, 8, then 200mg SC every 4 weeks) achieved 24.2% week 12 and 49.9% week 40 remission versus 13.3% and 25.1% placebo in LUCENT trials (19). Risankizumab (1200mg IV at weeks 0, 4, 8, then either 180mg SC every 8 weeks OR 360mg SC every 8 weeks) demonstrated 20.3% week 12 remission versus 6.2% placebo and week 52 remission of 40.2% (with 180mg Q8W) and 37.6% (with 360mg Q8W) versus 25.1% placebo in INSPIRE and COMMAND trials (21). Guselkumab (200mg IV at weeks 0, 4, 8, then 200mg SC every 4 weeks OR 100mg SC every 8 weeks) achieved 23% week 12 remission versus 8% placebo and week 44 remission of 50% (with 200mg Q4W) and 45% (with 100mg Q8W) versus 19% placebo in QUASAR trials, demonstrating among the highest remission rates of approved biologics for UC (13).

Next-generation anti-interleukin strategies employ innovative approaches: oral peptide IL-23 receptor antagonists with gut-selective blockade and picomolar affinity (icotrokinra), optimized IL-23p19 inhibitors (picankibart, brazikumab), selective gp130 trans-signaling inhibitors preserving classical IL-6 signaling (olamkicept), IL-4Rα antagonists for type 2-driven disease (dupilumab), IL-36 receptor antagonists (spesolimab), CD127/IL-7Rα antagonists (lusvertikimab), IL-1β neutralizing antibodies (lutikizumab), and IL-13-specific inhibitors (anrukinzumab, tralokinumab). The heterogeneity of UC pathogenesis suggests biomarker-driven patient selection based on mucosal cytokine expression, genetic polymorphisms, transcriptomic signatures, and inflammatory endotypes (Th17-high, type 2-high, neutrophil-predominant) may optimize therapy selection and address the concept of “molecular resistance” whereby prior therapies alter the mucosal immune landscape.

The following section reviews investigational anti-interleukin and anti-interleukin receptor antagonists currently in Phase II and III clinical development for moderate to severe UC.

#### 2.4.1 Icotrokinra

Icotrokinra is an investigational first-in-class targeted oral peptide that selectively blocks the IL-23 receptor with single-digit picomolar binding affinity. The ANTHEM-UC study (NCT06049017) is a Phase 2b multicenter, placebo-controlled, dose-ranging RCT evaluating three once-daily oral doses of icotrokinra versus placebo in 252 adults with moderately to severely active UC who had inadequate response or intolerance to conventional therapy, prior biologics, or JAK inhibitors, with the primary endpoint being clinical response at Week 12 (defined as ≥30% and ≥2-point decrease in mMS with rectal bleeding improvement). The study successfully met its primary endpoint with the highest dose achieving 63.5% clinical response versus 27% placebo at Week 12 and 30.2% clinical remission versus 11.1% placebo, with favorable tolerability and continued improvement through Week 28, though the study completion date and final comprehensive results are pending presentation at upcoming medical congresses.

#### 2.4.2 Picankibart

Picankibart (IBI112) is a recombinant anti-interleukin 23p19 subunit monoclonal antibody IgG1 for treating moderate to severe active UC. This multicenter, double-blind, placebo-controlled Phase 2 RCT (NCT05377580) enrolled 150 Chinese patients who were randomized to receive various doses of Picankibart through intravenous injection during the induction period (Period 1) followed by subcutaneous maintenance therapy (Period 2), with quadruple masking involving participants, care providers, investigators, and outcomes assessors. The primary endpoint measured the percentage of subjects achieving clinical remission at week 12, while secondary endpoints included clinical response rates, symptomatic relief, endoscopic remission, mucosal healing at weeks 12 and 52, plus quality of life assessments using IBDQ and SF-36 scores at multiple timepoints including weeks 24 and 64 during extension treatment.

Inclusion criteria required patients aged 18–75 years with UC diagnosis at least 3 months prior, moderate to severe disease defined as mMS ≥4 with MES ≥2, and prior treatment with at least one conventional therapy or first biological agent use, while excluding patients with CD, rectal-limited disease or colon involvement under 15cm and toxic megacolon. The study commenced July 2022 with primary completion in March 2024 and estimated overall completion by September 2025, conducted at the First Affiliated Hospital of Sun Yat-sen University in Guangzhou, demonstrating successful achievement of its primary efficacy endpoint in the induction Phase (62), as announced by the pharmaceutical company, while results are yet to be published.

#### 2.4.3 Brazikumab

Brazikumab is a fully human IgG2 monoclonal antibody that selectively targets the p19 subunit of interleukin-23. The primary study (NCT03616821) was a 54-week double-blind, placebo-controlled Phase 2 RCT evaluating brazikumab versus placebo in 242 participants with moderately to severely active UC, measuring clinical remission at week 10 as the primary endpoint along with safety parameters through week 68; the open-label extension (EXPEDITION OLE) (NCT04277546) was subsequent. Participants needed to be 18–80 years old with confirmed UC extending at least 15 cm from the rectum, inadequate response to conventional treatments, and negative pregnancy tests for women of childbearing potential, while excluding those with fulminant colitis, CD, recent biologic therapy, or significant comorbidities. The study was terminated in October 2023 due to the pharmaceutical company strategic decision to discontinue brazikumab development in IBD, despite earlier promising Phase 2 results in CD showing clinical response rates of 49.2% versus 26.7% with placebo at week 8.

#### 2.4.4 Tralokinumab

Tralokinumab (CAT-354) is a recombinant human monoclonal IgG4 antibody directed against IL-13 that was tested in a Phase IIa randomized, double-blind, placebo-controlled, parallel-arm, multicenter study (NCT01482884) evaluating its efficacy and safety as add-on therapy in patients with active, moderate-to-severe UC. The study enrolled 147 patients aged 18–75 years with diagnosed UC at least 90 days prior to randomization, total MS ≥6 including MES ≥2, who were non-hospitalized and treated with stable background UC therapy containing 5-aminosalicylates and/or low-dose glucocorticosteroids and/or purine analogues, with exclusions for pregnant/breastfeeding women, history of colostomy, current diagnosis of indeterminate colitis or CD, hepatitis B/C or HIV, and history of cancer.

The primary outcome was clinical response at week 8 defined as decrease in total MS of at least 3 points and 30% with decrease in RBS of at least 1 point or absolute RBS of 0 or 1, while secondary outcomes included clinical remission rate at week 8, mucosal healing rate at week 8, and changes from baseline in total MS, partial MS, modified Riley score, and various biomarkers including C-reactive protein, albumin, and calprotectin at multiple timepoints through week 24. The study was completed in June 2013 with actual primary completion in March 2013, showing that while the primary endpoint was not met with clinical response rates of 38% for tralokinumab versus 33% for placebo, there was a statistically significant improvement in clinical remission rates of 18% versus 6% respectively, suggesting potential therapeutic benefit in a subset of patients with moderate-to-severe UC (63).

#### 2.4.5 Dupilumab

Dupilumab, a humanized monoclonal IgG4 antibody that binds to the IL-4 receptor alpha subunit (IL-4Rα), thereby blocking both IL-4 and IL-13 signaling through type I and type II IL-4 receptor complexes to inhibit TH2-mediated allergic inflammation. It is being tested in this Phase 2 double-blind, placebo-controlled multicenter RCT (NCT05731128) to evaluate its efficacy and safety in participants aged 18 years and older with moderately to severely active UC that has an eosinophilic phenotype. The primary outcome measures the proportion of participants achieving clinical remission at Week 24 using mMS criteria, while secondary outcomes include clinical response rates, symptomatic remission, histologic-endoscopic healing, and safety parameters assessed through Week 52 with an optional open-label extension. Inclusion criteria require participants to have evidence of biomarker enrichment at screening, moderately to severely active UC with a baseline mMS of 5-9, inadequate response or intolerance to standard biologic therapy or conventional treatments, while excluding those with severe extensive colitis requiring hospitalization, UC limited to rectum only, presence of ostomy or fistula, history of eosinophilic colitis, or extensive colonic resection. The study began in January 2023 with an estimated primary completion date of June 2026 and final study completion in March 2027, enrolling an estimated 84 participants across 77 locations worldwide.

#### 2.4.6 Olamkicept

Olamkicept is a first-in-class gp130 trans-signaling inhibitor comprised of two complete extracellular domains of gp130 fused to the Fc region of human IgG1, selectively blocking the soluble IL-6 receptor/IL-6 complex while preserving classic IL-6 signaling. A double-blind, placebo-controlled Phase 2b RCT (NCT03235752) was completed in December 2020 and enrolled 91 adults with active UC across 22 clinical sites in East Asia, comparing biweekly intravenous infusions of olamkicept 600 mg or 300 mg versus placebo over 12 weeks, with the primary endpoint being clinical response defined as a decrease of 3 or greater and 30% or greater from baseline total MS plus at least 1-point reduction in RBS (64). The study completed in December 2020 and demonstrated that olamkicept 600 mg achieved significantly higher clinical response rates than placebo (58.6% vs 34.5%, adjusted difference 26.6%, P = 0.03), while the 300 mg dose showed no significant difference from placebo (43.3% vs 34.5%, P = 0.52), with treatment-related AEs occurring in 53.3% of 600 mg patients versus 50% in placebo, most commonly including bilirubin presence in urine, hyperuricemia, and increased aspartate aminotransferase levels.

#### 2.4.7 Lusvertikimab

Lusvertikimab (OSE-127) is a first-in-class monoclonal IgG4 antibody antagonist targeting the CD127 receptor (IL-7Rα) that was evaluated in the CoTikiS trial (NCT04882007), a Phase 2 multicenter, double-blind, placebo-controlled RCT with parallel assignment design conducted across 55 locations internationally. The study enrolled 136 adults aged 18–75 years with moderate to severe active UC (mMS 4-9) who had inadequate response to conventional therapies or failure/intolerance to biologics, with participants receiving three intravenous infusions at weeks 0, 2, and 6 of either placebo, 450mg, or 850mg doses (65, 66).

The primary outcome measured change in mMS from baseline to week 10, with key secondary endpoints including clinical remission rates, endoscopic improvement, and safety assessments, while inclusion criteria required confirmed UC diagnosis for at least 3 months with minimum 15cm extent from anal margin, specific RBS and SFS, and prior treatment history with corticosteroids, immunosuppressants, or biologics. The study completed in January 2025 after starting in October 2020, demonstrating significant efficacy with both doses achieving the primary endpoint of MS reduction compared to placebo, with the 450mg dose showing particularly strong results including 22% clinical remission versus 4.4% for placebo and 34.7% endoscopic remission versus 12.6% for placebo, while maintaining a favorable safety profile with only mild, transient lymphopenia as the most notable adverse event.

#### 2.4.8 Lutikizumab

Lutikizumab (ABT-981) is a humanized IgG4 monoclonal antibody that binds IL1β under investigation for UC in the Horizon trial (NCT06257875), a Phase 2 multicenter RCT comparing lutikizumab to adalimumab for induction and maintenance therapy in adults with moderately to severely active UC. The study includes participants aged 18 and older with a UC diagnosis for at least 90 days, active disease (mMS 5-9, MES ≥2), and inadequate response to conventional therapies, while excluding those with CD, disease limited to rectum, or prior adalimumab failure. The primary outcomes measure endoscopic improvement at Week 12 and AEs through Week 64, with the study beginning in March 2024, having enrolled 156 of the planned 200 participants across approximately 280 global sites. An interim analysis in July 2025 showed lutikizumab achieved numerically higher endoscopic improvement rates compared to adalimumab but failed to demonstrate sufficient differentiation for statistical significance, leading the pharmaceutical company to discontinue monotherapy development in UC.

### 2.5 Novel and emerging targets

The incomplete efficacy and variable durability of response observed with established therapeutic classes, including anti-TNF agents, integrin antagonists, IL-23 inhibitors, JAK inhibitors, and S1P modulators, has driven exploration of diverse novel mechanisms targeting previously unexploited pathways in UC pathogenesis (27). These investigational agents represent mechanistically heterogeneous approaches that can be conceptually organized into several categories: immune checkpoint modulation strategies targeting co-stimulatory and co-inhibitory pathways (PD-1 agonism with rosnilimab, CD40 antagonism with ravagalimab, CD28-CD80/86 blockade with abatacept, OX40 antagonism with KHK4083), which aim to restore immune homeostasis by modulating T cell activation thresholds; cell depletion approaches targeting pathogenic lymphocyte populations (anti-CD20 rituximab, anti-CD3 agents visilizumab and OKT3, anti IL7R lusvertikizumab); alternative cytokine and chemokine pathways including IP-10/CXCL10 antagonism (eldelumab), CCL11 blockade (bertilimumab), IL-22 agonism for epithelial repair (efmarodocokin alfa), IL-2 receptor modulation for Treg expansion (aldesleukin, NKTR-358, efavaleukin alfa), and oncostatin M receptor blockade (vixarelimab); the TL1A pathway representing one of the most compelling emerging targets based on strong genetic associations and early clinical efficacy with agents including tulisokibart, afimkibart, duvakitug, XmAb942, and the bispecific RO7837195; intracellular signaling inhibitors including RIPK1 kinase inhibitors targeting necroptosis pathways (GSK2982772, ABBV-668, eclitasertib/SAR443122), protein kinase C inhibition (sotrastaurin), SIK2/SIK3 inhibition (GLPG3970), NF-κB/IRF pathway modulation (CU104), MAP3K8/TPL2 inhibition (tilpisertib), DHODH inhibition (vidofludimus calcium), and PDE4 inhibition for intracellular cAMP elevation (apremilast, mufemilast, tetomilast, orismilast); tissue repair and barrier function enhancers including melanocortin-1 receptor agonism (PL-8177), LANCL2 agonism (omilancor), and NLRX1 agonism (amelenodor); protease inhibition with MMP9 antagonism (andecaliximab); and specialized approaches including TREM-1 antagonism (BI 3032950), TLR9 modulation (BL-7040), leukotriene pathway inhibition (LYS006), and antisense oligonucleotide strategies (mongersen targeting SMAD7). The remarkable diversity of these mechanisms reflects both the complex, redundant nature of inflammatory pathways in UC and the recognition that single-target approaches may have inherent efficacy limitations, potentially necessitating either highly selective patient stratification based on dominant pathogenic mechanisms or rational combination strategies targeting complementary pathways. The following sections review these novel and emerging therapeutic targets currently in Phase II and III clinical development for moderate to severe UC, organized by mechanism of action.

#### 2.5.1 PD1 agonists

##### 2.5.1.1 Rosnilimab

PD-1 is an inhibitory checkpoint receptor that normally dampens T cell responses, but in UC, dysregulated PD-1 signaling fails to adequately suppress pathogenic effector T cells, particularly PD-1high expressing T follicular helper and T peripheral helper cells that accumulate in inflamed mucosa. Rosnilimab is a novel PD-1 agonist IgG1 monoclonal antibody designed to reduce T cell proliferation and inflammatory cytokine secretion by selectively depleting PD-1high expressing T follicular helper, T peripheral helper, and T effector cells while sparing PD-1low cells. The ROSETTA study (NCT06127043) is a Phase 2, double-blind, placebo-controlled, parallel-group, multicenter RCT evaluating rosnilimab versus placebo in 132 subjects with moderate to severe UC, with primary outcomes focusing on efficacy, safety, tolerability, pharmacokinetics, pharmacodynamics, and immunogenicity. Inclusion criteria require patients aged 18 and older with clinical UC diagnosis, moderate to severe active disease (mMS ≥5 with MES ≥2), recent surveillance colonoscopy ruling out dysplasia or cancer, and history of inadequate response or intolerance to at least 2 UC therapy classes, while excluding those with CD, fulminant colitis, disease limited to rectum, prior colectomy plans, or previous PD-1/PD-L1 exposure. The study began in December 2023 with estimated completion in May 2026 and is currently actively recruiting across multiple international sites, with Phase 1 healthy volunteer data showing favorable safety profile, dose-proportional pharmacokinetics with 2-week half-life, and approximately 50% reduction in PD-1 expressing CD4+ and CD8+ T cells lasting over 30 days, supporting progression to this Phase 2 efficacy trial (67).

#### 2.5.2 TREM-1 antagonist

##### 2.5.2.1 BI 3032950

TREM-1 (Triggering Receptor Expressed on Myeloid cells-1) is expressed on myeloid cells and amplifies inflammatory responses by enhancing pro-inflammatory cytokine production and neutrophil activation. BI 3032950 is a TREM-1 antagonist currently in Phase 2 development for immunology applications, specifically being investigated for moderate to severe UC in trial (NCT06636656). The single-arm, open-label study enrolls 40 adults aged 18–80 who have inadequate response to prior biologics, with participants receiving intravenous induction therapy every 4 weeks for 12 weeks followed by subcutaneous maintenance injections every 4 weeks for up to 2 years. The primary outcome measures clinical remission using mMS at 12 weeks, while secondary endpoints include endoscopic remission, clinical response, and safety parameters, with the study having started in December 2024 and estimated completion in June 2028 across 52 locations in the US and Europe.

#### 2.5.3 Melanocortin-1 receptor agonist

##### 2.5.3.1 PL-8177

Melanocortin-1 receptor agonists exert anti-inflammatory and tissue-protective effects in the intestinal mucosa, with PL-8177 demonstrating promising clinical signals in early Phase trials. A Phase 2a RCT (NCT05466890) evaluated PL8177, an oral selective melanocortin-1 receptor agonist, against placebo in a 3:1 ratio for treating active UC over 8 weeks (68). The study enrolled 12 patients aged 18–75 with active UC defined by MES ≥2 and disease extending at least 5cm from the anal verge, excluding those with fulminant colitis, CD, recent immunosuppressive therapy, or significant comorbidities. The trial completed in February 2025 and demonstrated clinical remission in 33% of PL8177 patients versus 0% placebo, statistically significant clinical response in 78% versus 33% placebo (p<0.005), and symptomatic remission in 56% versus 33% placebo, with excellent safety profile and no treatment-related AEs reported, as announced by the pharmaceutical company. However, full results are yet to be published.

#### 2.5.4 CCL11 antagonist

##### 2.5.4.1 Bertilimumab

Bertilimumab is a recombinant human IgG4 monoclonal antibody that neutralizes human eotaxin-1 (CCL11), a chemokine involved in eosinophil recruitment and activation. A double-blind, placebo-controlled, parallel group multi-center Phase 2 RCT (NCT01671956) comparing bertilimumab 10 mg/kg versus matching placebo in a 2:1 ratio over a 4-week treatment period with three IV infusions at 2-week intervals, followed by 9 weeks of safety follow-up, enrolling an estimated 42 adult patients with active moderate to severe UC. The inclusion criteria required patients aged 18–70 years with diagnosed active moderate to severe UC for at least 3 months, MS of 6-12, MES ≥2, RBS ≥1, physician’s global assessment sub-score ≥2, and eotaxin-1 levels in colon tissue biopsies ≥100 pg/mg protein, while excluding patients with previous colonic surgery, current TPN, positive C. difficile, active TB, pregnancy, severe UC with systemic toxicity, recent immunosuppressive therapy, or TNFα antagonists use within 60 days. The study status remained as “recruiting” with unknown completion and no results posted as of the last verification in January 2018.

#### 2.5.5 NF-κB/IRF pathways

##### 2.5.5.1 CU104

CU104 is a new oral investigational drug that specifically inhibits IL1β, IL-6, and TNFα (69). It is being studied in a double-blind, placebo-controlled, multi-center Phase 2 RCT (NCT05907330) designed to evaluate its efficacy and safety in patients with moderate to severe UC. The study includes 45 participants aged 18–80 years who have active moderate to severe UC with a mMS of 5–9 and endoscopy sub-score of at least 2, excluding those with recent biologic treatments, extensive colonic resection, or other inflammatory bowel diseases. The primary outcome measures the percentage of participants achieving clinical remission (mMS 0-2) at Week 8, with the study estimated to complete by March 2026, though results are not yet available as the trial has not yet begun recruiting.

#### 2.5.6 Interleukin 2

##### 2.5.6.1 Aldesleukin

Low-dose IL-2 receptor agonists selectively expand and activate regulatory T cells that express high levels of CD25, aiming to restore immune homeostasis by enhancing immunosuppressive mechanisms that are deficient in UC. An open-label Phase 1b/2a trial (NCT02200445) investigated low-dose IL-2 (aldesleukin/Proleukin) as a T-cell growth factor designed to selectively expand regulatory T cells. The study used a 3 + 3 dose-escalation design with daily subcutaneous injections for 8 weeks across three cohorts (0.3, 1.0, and 1.5 million IU/m²/day) (70). Twenty-six adults aged 18–70 with moderate-to-severe UC (MS 6-10) were enrolled, all having failed at least one prior therapy and requiring stable concomitant medications. Exclusion criteria included CD, need for immediate intervention, prior colectomy, dysplasia, active infections, or significant laboratory abnormalities. Primary outcomes assessed safety and maximum tolerated dose, while secondary outcomes measured clinical response (≥3-point or ≥30% MS reduction) and remission (MS ≤2). The trial completed in March 2021 and identified 1.0 million IU/m²/day as the maximum efficacious dose. Among completers at this dose, 69% achieved clinical response and 31% achieved remission. Treatment was safe and well-tolerated aside from mild injection site reactions, fever, and malaise. The therapy successfully expanded peripheral regulatory T cells in nearly all patients regardless of clinical response. However, non-responders showed unwanted conventional T-cell activation, and mucosal regulatory T-cell expansion was neither necessary nor sufficient for clinical improvement.

#### 2.5.7 TL1A pathway inhibitors

##### 2.5.7.1 Tulisokibart

TL1A (tumor necrosis factor-like ligand 1A, encoded by TNFSF15) is a member of the TNF superfamily that signals through death receptor 3 (DR3) to promote T cell activation, proliferation, and inflammatory cytokine production (71). In UC, TL1A expression is markedly elevated in inflamed colonic mucosa and correlates with disease severity. Functionally, TL1A amplifies Th1 and Th17-driven inflammation by promoting expansion and maintenance of pathogenic effector T cells that produce IL-17A, IL-17F, IFN-γ, and other inflammatory mediators. The selective expression of TL1A and DR3 in inflamed intestinal tissue makes this pathway an attractive target for achieving gut-specific immunomodulation while minimizing systemic immunosuppression.

Tulisokibart (MK-7240) is a humanized IgG1 monoclonal antibody targeting TNFSF15/TL1A. The ARTEMIS-UC trial, a Phase 2 multicenter randomized double-blind placebo-controlled study, evaluated the efficacy and safety of tulisokibart as a 12-week induction treatment in patients with moderate-to-severe UC. Patients (n = 135) were randomized 1:1 to receive either placebo or tulisokibart administered intravenously (1000 mg on day 1, followed by 500 mg at weeks 2, 6, and 10). Primary endpoints included clinical remission (defined as RBS of 0 and SFS ≤1) and endoscopic improvement (defined as MES ≤1 with no friability). At week 12, clinical remission was achieved in 26.5% of patients in the tulisokibart group compared to 1.5% in the placebo group, while endoscopic improvement was observed in 36.8% versus 6% respectively. Additional benefits included reductions in bowel urgency, RBS, and SFS, with no serious adverse events occurring during the trial.

Following the 12-week induction period, participants could enter the ARTEMIS-UC extension study. Participants were classified as responders (defined as ≥2-point and ≥30% reduction in mMS from baseline, with ≥1-point reduction in RBS or absolute RBS ≤1 at week 12) or non-responders. Induction responders were randomized to receive tulisokibart at either 250 mg (n = 25) or 100 mg (n = 22). At week 50, induction responders maintained efficacy endpoints, with greater efficacy observed in the 250 mg group compared to the 100 mg group, while the drug continued to demonstrate a favorable safety profile.

The completed Phase 2 study (NCT04996797, ARTEMIS-UC) tested tulisokibart in 178 participants with moderately to severely active UC using a randomized double-blind placebo-controlled design with a companion diagnostic component. Participants aged 18 and older with corticosteroid dependence or treatment failures received the same intravenous dosing regimen described above. The study ran from July 2021 to July 2025 and achieved its primary endpoint with significantly higher clinical remission rates in the tulisokibart group compared to placebo (26% versus 1% in Cohort 1), with similar adverse event profiles between groups (72, 73).

A Phase 3 program (NCT06052059) is currently evaluating tulisokibart in participants with moderately to severely active UC through two separate randomized double-blind placebo-controlled studies with induction and maintenance components. The trial enrolls an estimated 1,020 participants aged 16–80 years who have had inadequate response, loss of response, or intolerance to conventional treatments including corticosteroids, immunosuppressants, or advanced therapies. Primary outcomes measure clinical remission according to mMS at week 12 for both studies and at week 52 for Study 1, with secondary outcomes including adverse events, clinical response rates, endoscopic improvement, and quality of life measures. The study began in October 2023 with primary completion estimated for November 2026 and final completion in December 2029.

An extension study (NCT06651281) is testing tulisokibart in participants who previously received the drug in parent studies for CD or UC. The trial uses a non-randomized parallel design with quadruple masking, enrolling an estimated 1,380 participants who must have participated in qualifying parent studies and shown clinical benefit. Primary outcomes measure adverse events and treatment discontinuations due to adverse events over approximately 378 weeks, with secondary outcomes assessing clinical remission rates at week 364. The study started in November 2024 with estimated completion in December 2037.

##### 2.5.7.2 Afimkibart

Afimkibart (PF-06480605/RO7790121) is a fully human IgG1 monoclonal antibody targeting tumor necrosis factor-like ligand 1A (TL1A) that neutralizes the TL1A-DR3 interaction and subsequent inflammatory signaling pathways (74). The clinical development program has progressed through multiple Phases demonstrating increasing efficacy in moderate to severe UC.

The Phase IIa TUSCANY trial (NCT02840721, 2016-2019) was a multicenter single-arm open-label study testing 500 mg intravenous doses every two weeks for seven total doses in 50 participants with moderate to severe UC. The study included adults aged 18–75 with active UC extending beyond the rectum (>15 cm), MS ≥6 with RBS ≥1 and MES ≥2, who had inadequate response to conventional therapies including steroids, immunosuppressants, TNFα antagonists, or anti-integrin inhibitors. Exclusion criteria included CD, dysplasia, active infections, or recent biologic use. The trial completed in August 2018 and demonstrated statistically significant endoscopic improvement in 38% of participants at week 14, endoscopic remission in 10%, and clinical remission in 24% (75). Additional benefits included reductions in fecal calprotectin and CRP levels. The study documented 109 treatment-emergent adverse events, with UC exacerbation and arthralgia being most common. A notable limitation was that 82% of patients developed anti-drug antibodies and 10% exhibited neutralizing antibodies, which could potentially compromise treatment efficacy and tolerability.

The Phase IIb TUSCANY-2 study (NCT04090411, 2019-2022) represented the pivotal dose-ranging trial conducted as a multicenter randomized double-blind placebo-controlled study. Enrolled participants (n = 246) underwent a 14-week induction period with different subcutaneous doses (50 mg, 150 mg, and 450 mg every four weeks), followed by a 56-week follow-up period. At week 14, endoscopic improvement was achieved in 36%, endoscopic remission in 11%, and clinical remission in 29% of patients. At week 56, these endpoints improved further with 50% achieving endoscopic improvement, 21% achieving endoscopic remission, and 36% achieving clinical remission. Robust histologic improvements were demonstrated with Geboes scores showing improvement in approximately 33-40% versus 12% placebo at week 14, sustained through week 56. The study showed early symptomatic benefits with rectal bleeding improvement visible by week 2 (34% achieved RBS of 0 versus 20% placebo) and symptomatic remission rates of 17.5% versus 8.9% placebo by week 2, indicating rapid onset of action (76). The drug was well tolerated across all dose groups and demonstrated a favorable safety profile.

Two Phase III trials are currently recruiting. Ametrine-2 (NCT06588855) is a multicenter double-blind placebo-controlled study evaluating 12-week induction therapy with afimkibart versus placebo in 350 participants with moderately to severely active UC, administered intravenously followed by subcutaneous injection. Ametrine-1 (NCT06589986) is a treat-through study examining both induction and maintenance therapy over 52 weeks in 400 participants. Both studies include participants aged 16–80 years with confirmed UC diagnosis, mMS indicating moderate to severe disease activity, body weight ≥40kg, adequate colorectal cancer screening, and demonstrated inadequate response or intolerance to at least one conventional or advanced UC therapy. Exclusion criteria include UC complications, other forms of colitis, ostomy, primary sclerosing cholangitis, pregnancy, recent malignancy, and specific infections including C. difficile, CMV, HIV, and hepatitis. The primary outcome for both studies is clinical remission defined as mMS ≤2 with specific SFS, RBS, and MES at week 12, with Ametrine-1 additionally measuring this endpoint at week 52. Secondary outcomes include endoscopic improvement and remission, clinical response, histologic improvement using Geboes scoring, changes in bowel urgency and abdominal pain, quality of life measures, and comprehensive safety assessments. Both studies began recruitment in late 2024 with Ametrine-2 primary completion estimated for June 2027 and study completion for both trials estimated for December 2029, recruiting across 152 locations worldwide including major medical centers in the United States, Europe, Asia, and other regions, though no results are yet available.

##### 2.5.7.3 Duvakitug

Duvakitug (TEV-48574) is a fully human IgG1 monoclonal antibody that targets TL1A-DR3 signaling to reduce inflammation and fibrosis in IBD patients, studied in the Phase 2b RELIEVE UCCD trial (NCT05499130) which randomized 290 adults with moderate to severe UC or CD to receive subcutaneous duvakitug (450mg or 900mg every 2 weeks after loading dose) or placebo for 14 weeks across 164 global sites. The primary endpoints were clinical remission for UC patients (mMS ≤2) and endoscopic response for CD patients (≥50% SES-CD reduction), with inclusion criteria requiring IBD diagnosis for ≥3 months, moderate to severe disease activity, and inadequate response to prior therapies, while excluding patients with active infections, malignancy, immunocompromised states, or pregnancy. The study completed in November 2024 demonstrating statistically significant efficacy with UC clinical remission rates of 36% (450mg) and 48% (900mg) versus 20% placebo, favorable safety profile with lower adverse event rates than placebo, and led to a long-term extension study (NCT05668013) providing 44-week maintenance therapy for responders and re-induction for non-responders to evaluate sustained efficacy and corticosteroid-free remission (77, 78).

##### 2.5.7.4 XmAb942

XmAb942 is an anti-TL1A IgG1 antibody with extended half-life technology. A Phase 1/2 randomized, double-blind, placebo-controlled study (NCT06619990) with single ascending doses in healthy volunteers (Part A), repeat dosing in healthy volunteers (Part B), and efficacy evaluation in moderate-to-severe UC patients (Part C), measuring primary outcomes of safety/tolerability and clinical remission via mMS over 12–28 weeks. Participants aged 18–55 with good health are included, with healthy volunteers in Parts A/B and UC patients in Part C, excluding those with significant medical conditions, cardiac issues, or other inflammatory bowel diseases, while the study began October 2024 with completion estimated for October 2027 (67).

##### 2.5.7.5 R07837195

RO7837195 is a p40xTL1A bispecific antibody developed through a Roche-Pfizer collaboration that simultaneously targets IL-12/IL-23 (via the p40 subunit) and TL1A pathways, currently being evaluated in the Phase IIb SUNCREST trial (NCT06979336) which employs a multicenter, double-blind, placebo-controlled design with a 12-week induction Phase followed by a 40-week active treatment extension in approximately 224 participants across five dosing arms. The study enrolls adults with moderately to severely active UC diagnosed for at least 3 months who have had inadequate response, loss of response, or intolerance to conventional therapy or biologic agents, while excluding those with prior extensive colonic resection, CD, or previous failure of TL1A or IL-12/IL-23 targeted therapies, measuring clinical remission at week 12 using mMS criteria as the primary endpoint alongside secondary outcomes including clinical response, endoscopic improvement, safety parameters, and pharmacokinetics. The trial is currently in the recruitment Phase with first patient enrollment expected in Q3–2025 and an estimated study completion date of October 2028, therefore no efficacy or safety results are yet available for this novel dual-pathway inhibitor that aims to overcome the therapeutic ceiling observed with single-target approaches in inflammatory bowel disease.

##### 2.5.7.6 ABBV-668

ABBV-668 is an investigational oral inhibitor of RIPK1 being developed by AbbVie for moderate to severe UC treatment. The Phase 2, single-arm, open-label trial (NCT05570006) enrolled 30 adult participants with moderate to severe UC (Adapted MS 5-9, MES ≥2) who had inadequate response to standard therapies, with participants receiving twice-daily oral capsules for 52 weeks followed by 30-day follow-up. The study completed in December 2024 with primary outcomes measuring endoscopic improvement at week 8 and AEs, though specific efficacy results have not yet been publicly reported, and subsequent reports indicate the IL-1 targeting drug may have encountered challenges in demonstrating clinical benefit.

##### 2.5.7.7 Eclitasertib

Eclitasertib (SAR443122) is a highly potent, selective oral RIPK1 kinase inhibitor that is been evaluated in the RESOLUTE trial (NCT05588843), a randomized, double-blind, placebo-controlled Phase 2 dose-finding study with 182 participants across four parallel arms testing three dose levels against placebo in adults with moderate to severe UC. The study consists of a 12-week induction Phase followed by a 40-week maintenance Phase, with the primary outcome measuring clinical remission at Week 12 using mMS, while secondary outcomes include endoscopic improvement, clinical response, histological improvement, and various patient-reported outcomes.

Participants must have active UC for at least 3 months with minimum 15cm disease extent from anal verge, inadequate response or intolerance to conventional treatments including amino-salicylates, corticosteroids, immunosuppressants or biologics, while excluding those with CD, indeterminate colitis, active infections, malignancies, or previous exposure to natalizumab or RIPK1 inhibitors. The study began in November 2022 with estimated primary completion in February 2026 and final completion in December 2026, currently recruiting across 105 locations in 22 countries including the United States, Europe, Asia, and Latin America, with no results yet available as the trial is ongoing.

#### 2.5.8 MiRNA upregulation

##### 2.5.8.1 Obefazimod

Obefazimod (ABX464) is an oral small-molecule drug that selectively upregulates miR-124 expression to reduce inflammation, developed for moderate to severe UC through a novel anti-inflammatory mechanism targeting pro-inflammatory cytokines like IL-17 and IL-6. The Phase 3 ABTECT program comprised two double-blind, placebo-controlled induction RCTs (NCT05507203 and NCT05507216) testing 25mg and 50mg once-daily doses versus placebo in 1,275 patients across 600+ sites in 36 countries, with the primary endpoint of clinical remission at week 8, enrolling patients with inadequate response to conventional or advanced therapies including the largest JAK inhibitor-experienced population in UC trials (79). The 50mg dose achieved the primary endpoint with pooled 16.4% placebo-adjusted clinical remission (p<0.0001), demonstrating 19.3% placebo-adjusted remission in ABTECT-1 and 13.4% in ABTECT-2, while meeting all key secondary endpoints including endoscopic improvement and clinical response, with favorable safety profile showing headache as the most common adverse event and no new safety signals, as the ongoing 44-week maintenance trial (NCT05535946) with 678 responders is expected to report Q2–2026 to support regulatory submissions planned for second half 2026.

#### 2.5.9 PDE4 inhibitors

##### 2.5.9.1 Apremilast

Apremilast is an oral phosphodiesterase 4 (PDE4) inhibitor that modulates inflammatory mediators intracellularly. A Phase 2 double-blind, placebo-controlled RCT (NCT02289417) enrolled 170 patients with active UC across three arms: apremilast 30mg twice daily, apremilast 40mg twice daily, or placebo for 12 weeks, followed by 40 weeks of active treatment and an optional 52-week extension Phase. The primary endpoint was clinical remission defined as total MS ≤2 with no individual subscore >1 at week 12, with secondary endpoints including clinical response, endoscopic remission, and safety measures.

Inclusion criteria required patients aged 18 or older with UC duration ≥3 months, total MS 6-11, MES ≥2, and therapeutic failure or intolerance to at least one conventional therapy including aminosalicylates, budesonide, corticosteroids, or immunosuppressants, while being naïve to biologic therapy. The study completed in June 2019 with results showing that 31.6% of patients in the 30mg group achieved clinical remission versus 12.1% in placebo (p=0.01), though the 40mg group showed only 21.8% remission (p=0.27 versus placebo), with both active groups demonstrating greater reductions in inflammatory markers and sustained remission rates up to 40% at week 52 (80).

##### 2.5.9.2 Mufemilast

Mufemilast (Hemay005) is a selective phosphodiesterase 4 inhibitor being tested in a multicenter, double-blind, placebo-controlled Phase II RCT (NCT05486104) with three arms comparing 45mg twice daily, 60mg twice daily, and placebo over 12 weeks of treatment followed by 4 weeks of observation. The study enrolled 92 patients across 18 centers in China and aims to evaluate clinical remission defined as MS of 0–1 in bowel movements with specific reductions from baseline, blood in stool score of 0, and endoscopic score of 0-1. Eligible participants are adults 18 years and older with moderate to severe UC extending beyond the rectum (≥15cm affected bowel segment) with mMS 4–9 points and MES ≥2 points, who have failed or shown intolerance to standard treatments including sulfasalazine, corticosteroids, immunosuppressants, or TNFα antagonists therapy. The study started in November 2022 with an estimated completion date of December 2025, and results are not yet available as the trial is ongoing but no longer recruiting participants.

##### 2.5.9.3 Orismilast

Orismilast (UNI-50001) is an oral B/D selective PDE-4 inhibitor being studied for moderate to severe UC in the UCORIS trial, a Phase 2a open-label single-arm exploratory study that enrolled adults with UC diagnosed for at least 3 months, MES of ≥2, on stable 5-ASA or immunomodulator therapy for 3 months, with treatment given twice daily for 12 weeks extendable to 52 weeks (81, 82). The primary endpoint was clinical remission defined as total MS ≤2 with no individual subscore >1 at week 12, and preliminary results from 9 enrolled patients showed 1 patient (11%) achieved the primary endpoint after 12 weeks while 2 additional patients achieved remission at 6 weeks but discontinued due to adverse effects (headache, nausea), with the most common side effects being nausea (67%), dizziness (22%), and headache (22%). The study began enrollment in January 2024 and is investigator-initiated at Hvidovre Hospital in Denmark with recruitment completed and preliminary data published in January 2025, showing modest efficacy signals with 3 of 9 patients (33%) achieving remission overall though tolerability concerns led to early discontinuation in several participants.

#### 2.5.10 NLRX1 agonist

##### 2.5.10.1 Amelenodor

Amelenodor is an oral triphenyl compound designed as a NLRX1 agonist that reduces intracellular reactive oxygen species and inflammation with gut-selective pharmacokinetics and minimal systemic absorption. A Phase 2 double-blind, placebo-controlled RCT (NCT05785715) tested 250mg and 750mg doses versus placebo in participants with moderate to severe UC, measuring clinical activity via mMS changes over 365 days with secondary safety endpoints including hematology, chemistry panels, and vital signs. Inclusion criteria required adults aged 18–75 with UC diagnosis over 90 days, total MS of 5 or higher, MES of 2 or higher, and RBS of 1 or higher, while excluding severe extensive colitis requiring hospitalization, fulminant colitis, CD, and recent toxic megacolon. The study was terminated early by sponsor decision (not safety-related) after completing enrollment of 81 participants in May 2025, though specific efficacy results from this Phase 2 trial are not yet published, with prior Phase 1b data showing the 250mg dose achieved 49% reduction in total MS and clinical response in 8/11 patients versus 0/4 on placebo.

#### 2.5.11 TLR9 modulation

##### 2.5.11.1 BL-7040

BL-7040, a synthetic oligonucleotide acting as a Toll-like receptor-9 (TLR9) modulator with dual nervous and immune system activity, was tested in an open-label Phase IIa multicenter trial (NCT01506362) with 22 enrolled patients receiving escalating oral doses (12mg daily for 19–21 days, then 40mg daily for 14 days) over 5 weeks. The study included adults aged 18–70 with moderately active UC (MS 5-9), MES ≥2, RBS ≥1, and disease extending ≥20cm from anal verge, while excluding those with CD, infections, or recent surgeries, with concomitant stable mesalamine and steroids permitted. The trial completed in May 2013 showed that 16 of 22 patients finished treatment with clinical remission achieved in 12.5% (2 patients), clinical response and mucosal healing in 50% of patients, significant reduction in mucosal neutrophils and IL-6 levels in responders, and good tolerability with only mild-to-moderate AEs and three discontinuations due to side effects (83).

#### 2.5.12 LANCL2 agonism

##### 2.5.12.1 Omilancor

LANCL2 is an intracellular receptor implicated in intestinal homeostasis, with LANCL2 agonism showing promising efficacy signals in Phase 2 trials with favorable safety and gut-restricted pharmacokinetics. Omilancor (BT-11), is a first-in-class, oral, gut-restricted LANCL2 agonist that has progressed through from Phase 1 safety studies in healthy volunteers to Phase 2 efficacy. A Phase 1 study in 70 healthy volunteers demonstrating excellent safety and tolerability up to 100 mg/kg daily doses with no dose-limiting toxicities, where it showed gut restriction with fecal concentrations 6000-fold higher than plasma levels and early biomarker signals including reduced fecal calprotectin. This was followed by the pivotal Phase 2 randomized, double-blind, placebo-controlled multicenter trial (NCT03861143) that enrolled 198 patients aged 18–75 with moderate UC (MS 4-10) across 16 sites in 11 countries, comparing Omilancor 440mg and 880mg doses against placebo once daily for 12 weeks with the primary endpoint of clinical remission defined by mMS criteria, which completed in June 2021. Among patients with active disease confirmed by rectal bleeding, histological activity, and elevated fecal calprotectin at baseline, omilancor achieved clinical remission in 30.4% of cases versus 3.7% with placebo, and endoscopic and histological remissions reached 41.7% in the omilancor group compared to 18.6% and 22.2% in placebo groups, respectively (84, 85). Phase III trials were announced by the pharmaceutical company, but have not yet been registered.

#### 2.5.13 Leukotriene pathway inhibition

##### 2.5.13.1 Lys006

Leukotriene A4 hydrolase (LTA4H) generates leukotriene B4, a potent chemoattractant for neutrophils that amplifies acute inflammation, and inhibiting this enzyme aims to reduce neutrophil-driven tissue damage in UC. LYS006 is a highly potent and selective LTA4H inhibitor developed as an oral capsule for treating neutrophil-driven inflammatory diseases. The study NCT04074590 was a randomized, double-blind, placebo-controlled Phase 2 proof-of-concept trial comparing LYS006 to placebo in a 2:1 ratio over 8 weeks in patients with mild to moderate UC, measuring clinical remission rate (MS ≤2 with no subscore >1) as the primary outcome and safety as secondary outcome. The study included adults aged 18–75 with established UC diagnosis for ≥3 months, active disease with MS 5-10, MES ≥2, and inadequate response to conventional 5-ASA therapy, while excluding those with CD, toxic megacolon, recent biologic treatments, or risk of colectomy. The trial was terminated early due to sponsor strategic considerations after enrolling only 23 of the planned participants, completing in November 2022, with results showing the compound was well-tolerated but efficacy data limited due to early termination and small sample size.

#### 2.5.14 MAP3K8/TPL2 inhibition

##### 2.5.14.1 Tilpisertib

MAP3K8 (also known as TPL2) is a serine/threonine kinase that regulates inflammatory responses downstream of TLRs and TNF receptors, controlling production of pro-inflammatory mediators including TNF-α, IL-1β, and COX-2 in UC. Tilpisertib (GS-4875) is an oral MAP3K8/TPL2 inhibitor tested in two Phase 2 trials for moderately to severely active UC. The first trial (NCT04130919) was a double-blinded, placebo-controlled RCT testing tilpisertib at 100mg and 300mg doses versus placebo, with the primary outcome being clinical remission per modified MS at Week 10, and secondary outcomes including endoscopic response, MS response and remission, and histologic remission via Geboes Scale; inclusion criteria required patients 18+ years with UC duration 3+ months, disease extent 15+ cm from anal verge, mMS 6–12 points, and prior inadequate response to TNFα antagonists, but this study was terminated early in February 2021 after enrolling only 19 participants because of a pharmaceutical company strategical decision. The second ongoing trial (NCT06029972, PALEKONA) is testing tilpisertib fosmecarbil (GS-5290), a prodrug formulation, in a dose-ranging study with three active doses versus placebo, targeting 176 participants with the primary endpoint being clinical response per modified MS at Week 12; inclusion criteria are similar but expanded to ages 18–75 years, requiring failure of at least one but no more than three advanced UC therapies, and this study began in December 2023 with estimated completion in 2027.

#### 2.5.15 Anti-CD3/CD3R

##### 2.5.15.1 Muronomab-CD3

CD3 is a core component of the T cell receptor complex, and oral anti-CD3 antibodies induce tolerogenic effects through T cell anergy, regulatory T cell induction, and anti-inflammatory cytokine production in gut-associated lymphoid tissue, though clinical development was limited by manufacturing discontinuation. Muromonab-CD3 (OKT3) is a murine anti-human IgG2a monoclonal antibody targeting the CD3 receptor complex on T lymphocytes, originally approved intravenously for preventing organ transplant rejection but repurposed for UC treatment with an oral version (86). An open-label pilot Phase 1b/2a trial (NCT01287195) administered oral OKT3 at 1–2 mg daily with omeprazole for 30 days to patients aged 18–65 with moderate-to-severe active UC (MS 6-12), excluding those on biologics or immunomodulators within four weeks. The study enrolled only 6 of the planned 16 participants between April 2011 and May 2013 due to early termination when the manufacturer discontinued OKT3 production. Primary outcomes measured safety through AEs monitoring, anti-drug antibody development, immune cell biomarker changes via flow cytometry, T-cell proliferation assays, and cytokine production in peripheral blood mononuclear cells, while secondary outcomes included clinical response using MS and endoscopic assessment. In patients who had taken oral OKT3, the drug was well-tolerated without serious treatment-related AEs or cytokine release syndrome, demonstrated biological activity through increased T-cell proliferation and anti-inflammatory gene expression changes, and achieved clinical response in 3 of 6 patients at week 3, though only modest endoscopic improvement occurred and effects appeared transient after treatment discontinuation (87).

## 3 Combination therapies

### 3.1 PF-06687234 and infliximab

A Phase 2a randomized, double-blind, placebo-controlled study (NCT03269695) evaluated PF-06687234, an investigational IL-10 fusion protein, as weekly subcutaneous add-on therapy to infliximab in patients with active UC who were not in remission despite stable infliximab treatment for at least 14 weeks. The primary outcome measured the percentage of participants achieving modified clinical remission at Week 12, defined as a modified total MS ≤2 with no individual subscore >1, traditional MES ≤1, and RBS = 0, while inclusion criteria required adults aged 18–75 years with active UC (total MS 4-9, MES ≥2) extending at least 15 cm from anal verge, on stable infliximab dosing 5–10 mg/kg every 6–8 weeks. The study was terminated early in January 2021 due to sponsor’s changed R&D strategy and priority, enrolling only 20 of the planned participants, with safety data showing treatment-emergent AEs but no efficacy results reported due to early termination and small sample size.

### 3.2 SPY001, SPY002, SPY003

A clinical trial investigating SPY001 SPY002, and SPY003 (long-acting IgG1 antibodies targeting α4β7, TL1A, and IL-23 respectively) both as monotherapies and in combinations for moderate to severe UC is currently undergoing. A Phase 2 multicenter platform trial with 645 participants aged 18 and older, featuring two parts (NCT07012395): Part A provides open-label safety and preliminary efficacy assessment of monotherapies, while Part B offers randomized, placebo-controlled evaluation of all six interventions (three monotherapies plus three combinations) compared to placebo. Inclusion criteria require confirmed UC diagnosis for at least 3 months with active disease extent of 15+ cm from anal verge, moderately to severely active disease defined by mMS 5–9 with RBS ≥1 and MES ≥2, while excluding current CD diagnosis, fulminant colitis requiring surgery, or failure of 4+ advanced therapy classes. The study began May 27, 2025 with estimated completion March 31, 2028, currently recruiting participants with primary outcomes measuring change in Robarts Histopathology Index for Part A and percentage achieving clinical remission for Part B; no results are yet available as the trial is ongoing.

### 3.3 Mirikizumab and tirzepatide

A study examines tirzepatide, a dual GLP-1/GIP receptor agonist administered subcutaneously, combined with mirikizumab, an IL-23 p19 antibody, in adults with moderately to severely active UC who are also obese or overweight (NCT06937086). It is a Phase 3b, randomized, multicenter, controlled study that began in June 2025 with an estimated completion date of April 2028. The study aims to enroll 350 participants across three treatment groups to determine whether combining mirikizumab with tirzepatide leads to both symptom reduction or resolution and at least 10% body weight loss compared to mirikizumab with placebo. Participants must have established UC for at least 3 months with moderate to severe activity (mMS 5-9), be obese (BMI ≥30) or overweight (BMI ≥27) with weight-related comorbidities, and have inadequate response to conventional or advanced therapies, while the study is currently actively recruiting with results not yet available.

### 3.4 Mirikizumab and eltrekibart

A Phase 2 adaptive dose-ranging study (NCT06598943) evaluates eltrekibart (LY3041658), a molecule that targets specific group of seven chemokines known as ELR+ CXC chemokines, which include CXCL1, CXCL2, CXCL3, CXCL5, CXCL6, CXCL7, and CXCL8. By neutralizing all seven of these chemokines, the antibody blocks their ability to signal through the CXCR1 and CXCR2 receptors. This study evaluates eltrekibart administered alone or in combination with mirikizumab for treating moderately to severely active UC in adults aged 18–75 who have inadequate response to conventional medications or advanced therapies. A randomized, double-blind, placebo-controlled trial measures clinical remission at 12 weeks as the primary outcome, with secondary endpoints including clinical response, endoscopic improvement and remission, and quality of life measures assessed through 52 weeks, requiring participants to have established UC diagnosis of at least 3 months duration while excluding those with prior anti-IL-23p19 or anti-IL-12p40 antibody exposure. The study began enrolment in October 2024 with an estimated 140 participants across 204 global locations, with primary completion expected in December 2027 and final study completion in September 2028, though results are not yet available as the trial is currently in the recruitment Phase.

### 3.5 Guselkumab and golimumab

A Phase 2a proof-of-concept trial tested guselkumab plus golimumab combination therapy against individual monotherapies in 214 patients with moderately to severely active UC aged 18-65 (VEGA Study, NCT03662542). The randomized, double-blind study enrolled participants with confirmed UC diagnosis of at least 3 months and MS of 6-12, excluding those with severe extensive colitis or rectum-limited disease. The study ran from November 2018 to November 2021, with primary endpoint of clinical response at week 12 defined as 30% MS reduction plus 3-point absolute decrease with rectal bleeding improvement. Results showed combination therapy achieved 83% clinical response versus 61% for golimumab monotherapy and 75% for guselkumab monotherapy, with AEs including UC flares, respiratory infections, and headache but no deaths or malignancies during the 12-week induction period, demonstrating superior efficacy of dual biologic approach over single-agent treatment (20).

An ongoing Phase 2b randomized, double-blind trial investigates the golimumab/guselkumab combination therapy (DUET-UC Study, NCT05242484) at various dose levels compared to placebo and individual monotherapies in 577 patients with moderately to severely active UC who failed prior advanced therapies. The study enrolled participants aged 18–65 with confirmed UC for at least 3 months, excluding those with severe extensive colitis, rectum-limited disease, or recent malignancy, and ran from September 2022 to May 2025 for primary completion with final study completion estimated for March 2029. Primary outcome measures clinical remission at week 48 based on modified Mayo subscores, with secondary endpoints including endoscopic improvement, corticosteroid-free remission, and comprehensive safety assessments including AEs, laboratory parameters, and immunogenicity through week 48.

Interestingly, the first controlled trial of SOR102, an oral bispecific antibody targeting both TNF and IL-23p19 in UC was recently published (88). It demonstrated a tolerable safety profile with minimal systemic exposure and preliminary efficacy signals exceeding 50% clinical response in the twice-daily treatment group.

### 3.6 OD-07656 and vedolizumab

OD-07656 is a small molecule inhibitor that acts on IL-1 receptor-associated kinase 4 (IRAK4), an essential enzyme within the toll-like receptor (TLR) signaling cascade that plays a central role in innate immune responses and mediates signaling through the TLR/IL-1β axis (89). IRAK4 functions as a critical kinase positioned downstream in the TLR pathway, where it serves a positive regulatory function in signal transduction. Upon recognition of pathogen-associated molecular patterns, TLRs initiate IRAK4 activation through the recruitment of MyD88, which binds to the Toll/IL-1 receptor domain. Once activated, IRAK4 phosphorylates and activates IRAK1 while simultaneously undergoing autophosphorylation, triggering a signaling cascade that culminates in NF-κB activation. This transcription factor subsequently upregulates the expression of various inflammatory mediators, thereby amplifying the inflammatory response.

A Phase 2a trial (NCT06850727) organized as a two-part, open-label and randomized study evaluating the safety and efficacy of OD-07656 followed by vedolizumab therapy in patients with moderately to severely active UC, with primary outcomes measuring changes in the 3-component mMS from baseline to 12 weeks and treatment-emergent AEs. The study includes adults aged 18–75 years with confirmed UC diagnosis who have had inadequate response to standard therapies including aminosalicylates, corticosteroids, immunosuppressants, TNFα antagonists, anti-IL-12/23 biologics, JAK inhibitors, or S1P modulators, while excluding patients with CD, extensive colonic resection, or colostomy/ileostomy. The trial started in June 2025 with an estimated enrollment of 57 participants across locations in Australia, Moldova, and New Zealand, with primary completion estimated for January 2026 and full study completion by November 2026, though results are not yet available as the study is currently in the recruiting Phase.

### 3.7 Infliximab and ustekinumab

A Phase 2 trial (NCT06453317) compares infliximab and ustekinumab monoclonal antibodies individually versus their combination in treating moderate to severe UC, with the primary outcome measuring clinical and endoscopic remission rates after induction therapy at weeks 14-16. The study includes 172 adult patients aged 18–65 with UC for at least 3 months who have inadequate response to standard treatments including corticosteroids and immunosuppressants, while excluding those with previous biologic use, severe comorbidities, or active infections. The trial began in February 2025 with estimated completion in June 2028, and no results are currently available as the study is actively recruiting participants in Poland.

## 4 Future directions and conclusions

The therapeutic landscape for moderate to severe UC is undergoing transformative evolution, with over 100 investigational agents targeting diverse inflammatory pathways currently in phase 2 and 3 development. This unprecedented pipeline offers genuine opportunities to overcome the therapeutic ceiling that has constrained long-term remission rates below 40-50% with current advanced therapies.

Several mechanistic classes demonstrate particular promise. TL1A pathway inhibitors represent the most compelling novel target, with phase 2 data showing clinical remission rates exceeding 25% versus less than 2% for placebo across both treatment-naïve and treatment-experienced populations. Next-generation selective JAK1 and TYK2 inhibitors aim to preserve efficacy while mitigating safety concerns through enhanced selectivity. Gut-restricted therapies including obefazimod, MORF-057, and OST-122 achieve therapeutically relevant tissue concentrations with minimal systemic exposure, potentially optimizing the benefit-risk ratio. Combination therapy strategies may prove essential for breaking the therapeutic ceiling, with dual biologic approaches demonstrating 83% clinical response versus 61-75% for monotherapy in controlled trials. Additional promising mechanisms targeting immune checkpoints, RIPK1-mediated necroptosis, and tissue repair pathways offer complementary approaches for patients failing conventional treatments.

Critical challenges remain that will determine which agents successfully translate to clinical practice. High phase 2 and 3 failure rates underscore the persistent difficulty of converting mechanistic rationale into clinical efficacy. The absence of validated predictive biomarkers limits precision medicine approaches, though companion diagnostics for TL1A and prognostic transcriptional signatures are under investigation. Innovative trial methodologies including exposure-driven dosing, target engagement confirmation, adaptive Bayesian designs, and master protocols can improve development efficiency while reducing participant burden (90). The integration of objective qualification criteria, extended induction periods, and head-to-head comparisons in late-phase trials provides increasingly clinically relevant efficacy data that better informs treatment decisions. The high attrition rate observed in UC drug development reflects not only scientific challenges but also evolving ethical considerations. As multiple effective therapies have become licensed for moderate-to-severe UC, the appropriateness of placebo-controlled trials has been questioned by many clinicians and patient advocates. This ethical concern may contribute to recruitment difficulties and higher dropout rates in placebo arms, particularly among patients who have access to approved alternatives. Active-comparator designs and innovative trial methodologies, such as adaptive platform trials and treat-through designs, represent potential solutions that may better balance scientific rigor with ethical patient care.

The convergence of mechanistically diverse therapeutics, biomarker-guided patient selection, rational combination strategies, and advanced trial designs positions the field for a paradigm shift toward precision medicine in UC. Success will require collaborative efforts to develop and validate predictive biomarkers, establish optimal treatment sequencing algorithms, evaluate long-term safety of novel agents and combinations, and demonstrate meaningful impact on patient-centered outcomes including hospitalization, surgery, disability, and quality of life. The breadth and mechanistic diversity of the current pipeline, coupled with methodological innovations in clinical trial design, suggest that achieving durable deep remission for the majority of patients with moderate to severe UC may be attainable within the next decade.
